# Phiclust: a clusterability measure for single-cell transcriptomics reveals phenotypic subpopulations

**DOI:** 10.1186/s13059-021-02590-x

**Published:** 2022-01-10

**Authors:** Maria Mircea, Mazène Hochane, Xueying Fan, Susana M. Chuva de Sousa Lopes, Diego Garlaschelli, Stefan Semrau

**Affiliations:** 1 Leiden Institute of Physics, Leiden University, Leiden, The Netherlands; 2 Leiden Academic Center for Drug Research, Leiden University, Leiden, The Netherlands; 3 Department of Anatomy and Embryology, Leiden University Medical Center, Leiden, The Netherlands; 4 Networks Unit, IMT School for Advanced Studies, Lucca, Italy

**Keywords:** Clusterability, scRNA-seq, Random matrix theory

## Abstract

The ability to discover new cell phenotypes by unsupervised clustering of single-cell transcriptomes has revolutionized biology. Currently, there is no principled way to decide whether a cluster of cells contains meaningful subpopulations that should be further resolved. Here, we present phiclust (ϕ_*clust*_), a clusterability measure derived from random matrix theory that can be used to identify cell clusters with non-random substructure, testably leading to the discovery of previously overlooked phenotypes.

**Supplementary Information:**

The online version contains supplementary material available at 10.1186/s13059-021-02590-x.

## Background

Unsupervised clustering methods [1–4] are integral to most single-cell RNA-sequencing (scRNA-seq) analysis pipelines [5], as they can reveal distinct cell phenotypes. Importantly, all existing clustering algorithms have adjustable parameters that have to be chosen carefully to reveal the true biological structure of the data. If the data is over-clustered, many clusters are driven purely by technical noise and do not reflect distinct biological states. If the data is under-clustered, subtly distinct phenotypes might be grouped with others and will thus be overlooked. Furthermore, most analysis pipelines rely on qualitative assessment of clusters based on prior knowledge, which can hinder the discovery of new phenotypes.

To assess the quality of a clustering quantitatively and help choose optimal parameters, some measures of clustering quality and clusterability have been proposed [6, 7], most of which are not directly applicable to scRNA-seq data. For example, some existing methods rely on multimodality of the expression matrix, which is not always justified for scRNA-seq data, especially when considering highly dynamic systems. Other methods have input parameters, such as the optimal number of dimensions for dimensionality reduction, that cannot be easily determined. Also, general methods do not explicitly account for uninformative sources of variability, related to cell cycle progression or the stress response, for example, which can be important confounders. In the context of scRNA-seq, one of the most widely used measures is the silhouette coefficient [8]. This measure requires the choice of a distance metric to compute the similarity between cells. Notwithstanding its usefulness, it cannot be excluded that a partition of random noise obtains a high silhouette coefficient, indicating high clustering quality. Other measures based on distance metrics or the fit of probability densities suffer from similar issues and often only provide binary results instead of a quantitative score [7]. A different approach is pursued by ROGUE [9], a recently developed tool to assess clustering quality specifically in scRNA-seq data. ROGUE applies the concept of entropy on a per-gene basis to quantify the mixing of cell types. While a clear improvement over existing methods, ROGUE depends on a challenging step of selecting informative genes to explain the differences between cell types. It also assumes a particular noise distribution and requires the careful choice of an adjustable parameter.

Here, we present phiclust (ϕ_*clust*_), a new clusterability measure for scRNA-seq data that addresses some of the shortcomings of existing methods. This measure is based on the angle *ϕ* between vectors representing the noise-free signal and the measured, noisy signal, respectively. We consider clusterability to be the theoretically achievable agreement with the unknown ground truth clustering, for a given signal-to-noise ratio. (Below, we will describe in detail how we define “signal” and “noise” in this context.) Importantly, our measure can estimate the level of achievable agreement without knowledge of the ground truth. High clusterability (phiclust close to 1) means that multiple phenotypic subpopulations are present and that clustering algorithms should be able to distinguish them. Low clusterability (phiclust close to 0) means that the noise is too strong for even the best possible clustering algorithm to find any clusters accurately. If phiclust equals 0, the observed variability within a cluster is consistent with random noise. Any subclusters of such a cluster still have a phiclust of 0, which prevents over-clustering of random noise. Instead of assuming a certain noise distribution or relying on a selection of informative genes, our measure can be applied to arbitrary types of random noise and includes all genes in the analysis. This is made possible by certain universal properties of random matrix theory (RMT) [10], which has been applied successfully in finance [11], physics [12] and recently also scRNA-seq data analysis [13].

Below, we will use results of RMT on the singular value decomposition (SVD) of a single-cell gene expression matrix, where rows correspond to genes and columns correspond to cells. To get an intuitive understanding of RMT, it is useful to first consider the cell-cell correlation matrix, calculated from the gene expression profiles. We start from the null hypothesis that the data does not contain any structure and is produced by a random process. In the context of single-cell transcriptomics, “structure” means multiple, distinguishable clusters of cells, or phenotypes. RMT can predict, what the correlation matrix looks like, if the entries of the gene expression matrix are samples of random variables that are independent and identically distributed. Trivially, the diagonal elements of this correlation matrix are all equal to 1. The off-diagonal elements are not exactly 0, however, despite the absence of any meaningful structure in the data. Only in the limit of measuring an infinite number of (random) genes would the off-diagonal elements become identically 0, and the correlation matrix would become the identity matrix. In that case, the only eigenvalue of the correlation matrix is 1. RMT describes the properties of a correlation matrix for a finite ratio of cells and genes. These correlation matrices are, in a sense, distributed “around” the identity matrix, which corresponds to an eigenvalue spectrum distributed around 1. Although the individual entries of the correlation matrix fluctuate from realization to realization, RMT shows that the eigenvalue spectrum is robust (a so-called “self-averaging” property) and an analytical expression for it can be obtained [14]. Likewise, RMT predicts that the singular value distribution of a purely random matrix is closely approximated by the Marchenko-Pastur (MP) distribution. This result holds true irrespective of the distribution of the random variable. This universal property of random matrices allows us to apply RMT to gene expression matrices obtained by scRNA-seq. Of course, any biologically interesting scRNA-seq measurement should contain structure, usually in the form of cell clusters. RMT allows us to regard singular values lying above the MP distribution as evidence for the rejection of the null hypothesis (i.e., the absence of structure in the data). The MP distribution is characterized by sharp upper and lower limits for the singular values of a random matrix but is strictly valid only in the limit of infinite numbers of genes and cells (while keeping the cell-gene ratio fixed). For finite matrices, the largest and smallest singular values are distributed around those sharp limits, which is described by the Tracy-Widom distribution [15].

**Fig. 1:**
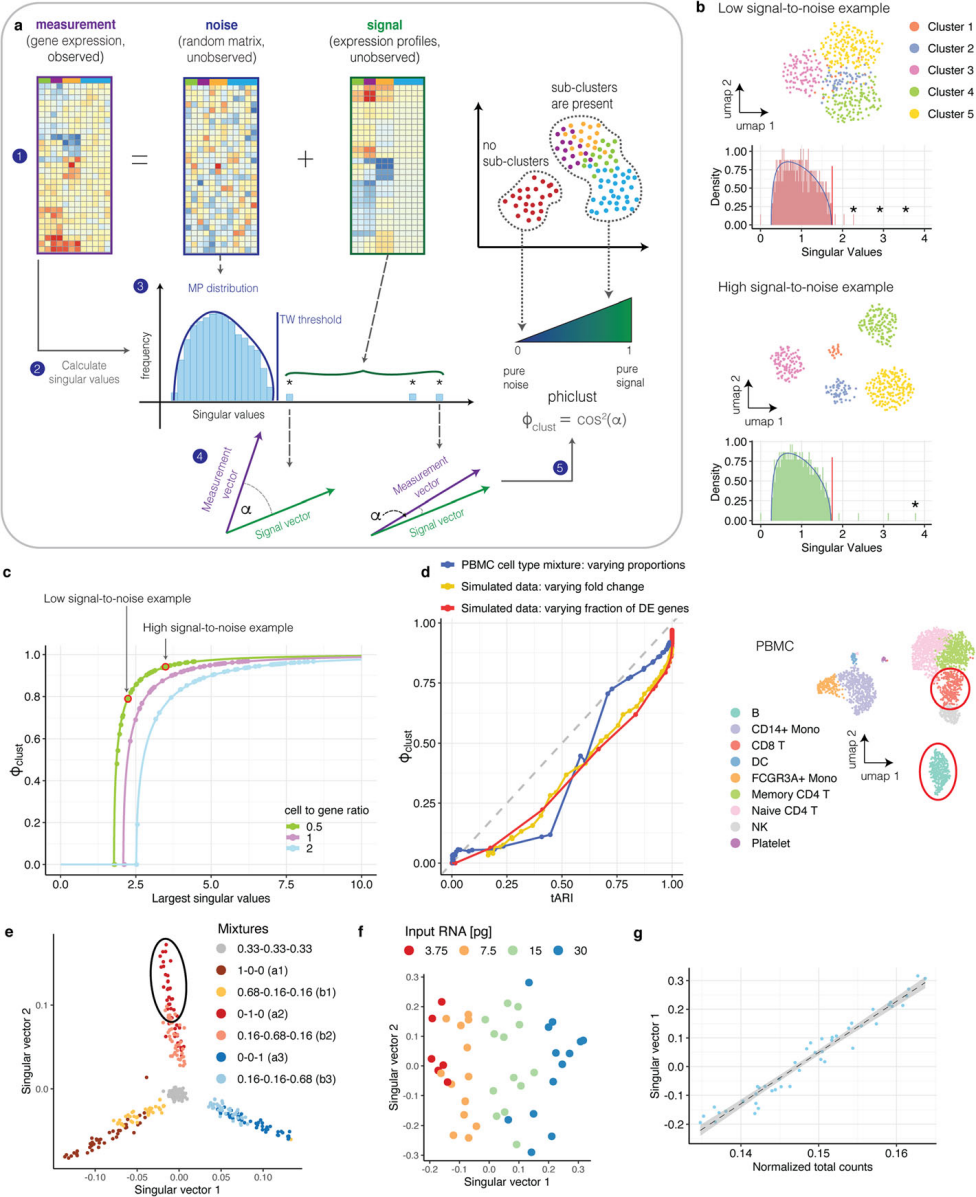
Phiclust is a proxy for the theoretically achievable adjusted rand index (tARI). **a** Scheme illustrating the rationale behind phiclust. **b** Singular value distributions of simulated data sets with 5 clusters and different levels of noise; Red: low signal-to-noise, Green: high signal-to-noise. The MP distribution is indicated by a solid blue line, the TW threshold is indicated by a red solid line, and significant singular values are highlighted with asterisks. Inserts show UMAPs of the data. The data set with a higher signal-to-noise ratio has more significant singular values and those singular values are bigger. **c** Value of the largest singular value versus for simulated data. Arrows indicate where the examples from **b** are located. The relationship between the largest singular values and phiclust only depends on the dimensions of the expression matrix. Simulations with different cell-to-gene ratios are shown in different colors. **d** Phiclust versus theoretically achievable ARI (tARI). Red data points: simulated data sets with two clusters. The number of differentially expressed (DE) genes was varied; the log fold change between clusters was fixed. Green data points: simulated data sets with two clusters. The mean log fold change between clusters was varied; the number of differentially expressed genes was fixed. Blue data points: two synthetic clusters were created by weighted averages of cells from two clusters in the PBMC data set. Cluster weights were varied. The grey dashed line indicates identity. Inset: UMAP of PBMC data set with the two clusters used indicated by red solid circles. **e** scRNA-seq of mixtures of RNA extracted from three different cell lines. Each data point is a mixture. For each mixture, the entries of the first two singular vectors are plotted. Colors indicate different ratios of contributions from the three cell lines. **f** First two singular vectors of the cluster indicated by a black solid ellipse in **e**. The amount of mRNA per mixture [pg] is indicated in color. **g** Normalized total counts per mixture versus first singular vector of the cluster shown in **f**. Linear regression (dashed line) is used to regress out the correlation with the total counts. Grey area indicates standard deviation

As explained above, the presence of structure manifests itself as singular values above the MP distribution (i.e., the prediction for a purely random matrix). Qualitatively, the magnitude of those outlying singular values corresponds to the magnitude of the differences between clusters. We can understand this relationship, if we assume that the measured gene expression matrix is the sum of a random matrix (the “noise”) and a matrix of noise-free gene expression profiles (the “signal”); see Fig. 1a. The bigger the difference in gene expression between phenotypes, the larger the magnitude of the non-zero singular values of the signal matrix. If the number of non-zero singular values (i.e., the rank of the signal matrix) is small compared to the dimensions of the matrix, low-rank perturbation theory [16] is applicable. This theory allows us to calculate the singular values of the measured gene expression matrix from the singular values of the signal matrix. Remarkably, knowledge of the complete signal matrix is not required for this calculation.

phiclust is meant to help identify non-random (or deterministic) structure. At the level of a complete data set, for example of a complex tissue, clusters are typically easily discernible. However, if we zoom in on a single cluster, it is much more difficult to decide, whether the variability within that cluster corresponds to meaningful sub-structure (such as the presence of multiple phenotypes) or is consistent with random noise. Below, we will precisely define a notion of clusterability, based on the adjusted rand index, and show that it strongly correlates with phiclust. Furthermore, we will demonstrate that our measure compares favorably to the silhouette coefficient and ROGUE on simulated data and experimental data sets with known ground truth. (See Table S1 for a list of all used simulated and experimental data sets.) Finally, we will apply phiclust to scRNA-seq measurements of complex tissues and obtain new biological insights, which we validate with follow-up measurements.

## Results

### Phiclust is derived from first principles and does not have free parameters

To derive phiclust, we considered the measured gene expression matrix as a random matrix perturbed by the unobserved, noise-free gene expression profiles (Fig. 1a). This is the exact opposite of the conventional approach, which considers random noise as a perturbation to a deterministic signal. Note that, in our approach, the random matrix contains both the biological variability within a phenotype as well as the technical variability (which is due to limited RNA capture and conversion efficiency, for example). Our point of view allows us to leverage well-established results from RMT [13, 17] and perturbation theory [16].

Figure S1 illustrates the basic principles that were applied to derive phiclust. RMT predicts that the SVD of a random noise matrix results in normal distributed singular vectors and a distribution of singular values that is closely approximated by the MP distribution, if the matrix is large enough (Additional file 1: Fig. S1a, left column). Here, we consider the noise-free gene expression profiles of the cells in various phenotypes, as the “signal” that perturbs the random matrix and thus its singular value distribution. Since biological and technical variability are lumped into the random matrix, expression profiles are identical for cells that belong to the same phenotype. For example, in the case of two distinct phenotypes, the signal matrix has only one non-zero singular value (Additional file 1: Fig. S1a, middle column). The observed (or measured) gene expression matrix is obtained as the sum of the random noise matrix and the noise-free gene expression profiles (Additional file 1: Fig. S1a, right column). The singular value distribution of the measured expression matrix has exactly one singular value above the upper limit that the theory predicts for a purely random matrix, the Tracy-Widom (TW) threshold. The outlying singular value and its associated singular vector correspond to the deterministic component of the measured expression matrix. The distribution of the remaining singular values (the “bulk”) is still closely approximated by the MP distribution. Importantly, as the perturbation becomes larger, the value of the outlying singular value also increases (Additional file 1: Fig. S1b). A larger perturbation means more distinct and therefore more easily clusterable phenotypes (compare the singular vectors in the middle row of Figs. S1a and b). The basic idea of phiclust is to use the magnitude of the outlying singular values to quantify clusterability.

Due to the universality of RMT, all described principles are independent of the particular noise distribution (see Additional file 1: Fig. S1a-b for normal distributed noise and Additional file 1: Fig. S1c-d for Poisson distributed noise). SVD of appropriately preprocessed real data sets therefore leads to singular value distributions with the same shape as obtained in simulations: a bulk closely approximated by the MP distribution and one or multiple outlying values. We found that data preprocessing has to comprise normalization and log-transformation, as well as gene-wise and cell-wise scaling (Additional file 1: Fig. S2a-d). SVD of raw data or log-transformed, normalized data typically results in a largest outlying singular value that is much larger than all others (Additional file 1: Fig. S2a,b). The corresponding singular vector reflects a global trend in the data and is called “market mode” in the context of stock market analysis [11, 18]. Here, we call it “transcriptome mode”, since it corresponds to an expression trend that is present across all cells, irrespective of cell type (such as, for example, high expression of particular cytoskeletal genes or essential enzymes and low expression of certain membrane receptors or transcription factors). The transcriptome mode is obviously not informative for clustering. Scaling shifts its singular value to 0, which effectively removes it from further analysis (Additional file 1: Fig. S2c,d). We tested for all data sets used in this study, whether the bulk of the singular value distribution of each cluster deviates significantly from the MP distribution after the described preprocessing (Kolmogorov-Smirnov test, Additional file 1: Fig. S2e). For reasonably large clusters (> 50 cells), we only found one example of a (marginally significant) deviation from the MP distribution.

We next wanted to confirm, for real data, that the remaining outlying singular values reflect the strength of the signal, i.e., differences between the phenotypes. To that end, we extracted the gene expression profiles from two clusters in an experimental single-cell RNA-seq data set and added, as additional signal, a matrix with one non-zero singular value. As to be expected, SVD of the combined data results in one additional singular value, which increases with the strength of the perturbation (Additional file 1: Fig. S2f-g). See Table S2 for a list of all outlying singular values of experimentally measured expression matrices as well as the corresponding signal matrices. All in all, these tests show that the basic principles of random matrix theory and perturbation theory are applicable to real single-cell RNA-seq data.

So far, we have shown that the values of the outlying singular values are, qualitatively, related to the differences between phenotypes. However, their magnitudes are difficult to interpret. Phiclust is derived from the outlying singular values and can be interpreted as a measure of clusterability, as we will show in the next section. More specifically, phiclust is defined as the squared cosine of the angle between the leading singular vector of the measured gene expression matrix and the corresponding singular vector of the unobserved, noise-free expression matrix. Low-rank perturbation theory is able to predict this angle using only the dimensions of the measured gene expression matrix and its singular values, but without knowledge of the noise-free expression profiles. See Additional file 2 for a detailed derivation. If the noise level is low compared to the signal, this angle will be small, since the measured gene expression matrix is then very similar to the noise-free signal. This would result in phiclust close to 1. As the level of noise increases, for a fixed signal, the singular vectors of the measured expression matrix and the noise-free signal become increasingly orthogonal and phiclust approaches 0. To illustrate the calculation of phiclust, we simulated data sets with realistic noise structure using the *Splatter* package [19] (Fig. 1b,c). As to be expected, increasing the number of genes that are differentially expressed between clusters makes the clusters more easily separable and leads to larger singular values outside of the MP distribution (Fig. 1b). By construction, this results in higher values of phiclust (Fig. 1c). Please refer to Table S2 for the numerical values of the outlying singular values in the simulated expression matrices as well as the corresponding signal matrix.

We would like to stress at this point that phiclust is derived from universal properties of perturbed random matrices, which can be considered first principles. By contrast, many other measures are developed based on empirical observations and justified post hoc by their usefulness. Phiclust is calculated using only the SVD and the dimensions of the expression matrix. Thus, it does not have any free, adjustable parameters, which would have to be chosen by the user or learned from the data.

### Phiclust is a proxy for clusterability

To show that phiclust is a proxy for clusterability, we have to make the concept of clusterability more precise and quantifiable. We adopted the Adjusted Rand Index (ARI) [20] as a well-established measure for the agreement between an empirically obtained clustering and the ground truth. Next, we will argue that perfect agreement with the ground truth (ARI = 1) is not achievable in the presence of noise, even with the best conceivable clustering algorithm.

Take, for instance, the simplest possible case of two cell types, A and B. Without any noise (technical or biological), expression profiles within a cell type are identical and the data can be clustered perfectly. Correspondingly, the singular vector of the expression matrix has only two different entries (Additional file 1: Fig. S3a, left). Therefore, it is easy to find a threshold that discriminates between the two cell types. In the presence of noise, however, there is a chance that the measured expression profile of a cell from cell type A looks more like cell type B and is therefore clustered with other cells from cell type B and vice versa. Correspondingly, the entries of the singular vector are now spread by the noise and can overlap (Additional file 1: Fig. S3a, right). Even if we use the best possible threshold to discriminate between the two cell types, some cells will be necessarily misclassified, if the distributions overlap.

This type of error is unavoidable (or irreducible) and known as Bayes error rate [21] in the context of statistical classification. From the overlap of the singular vector entries, we can calculate the Bayes error rate or, equivalently, a theoretically achievable ARI (tARI, see also Additional file 2). Of course, this is only possible for data with known ground truth. We first used simulated data to show empirically that commonly used clustering methods are not able to exceed the tARI (Additional file 1: Fig. S3b,c). It therefore quantifies our notion of clusterability: With increased noise, tARI decreases and it is more challenging even for the best conceivable clustering algorithm to distinguish the difference between phenotypes. Importantly, phiclust is strongly correlated with the tARI (Fig. 1d) and thus allows us to estimate clusterability without knowing the ground truth.

So far, we have assumed additive noise (i.e., the measured gene expression is the sum of a random matrix and the noise-free expression matrix). Low-rank perturbation theory also makes a prediction for multiplicative noise (i.e., the measured gene expression is the product of a random matrix and the noise-free expression matrix). In that case, phiclust still scales approximately linearly with the tARI, but its dynamic range depends somewhat on the cell-to-gene ratio (Additional file 1: Fig. S3d). To our knowledge, the noise generating mechanisms at work in scRNA-seq have not been pinpointed comprehensively. Therefore, we will continue to assume additive noise, noting that our approach can be easily adapted to multiplicative noise.

To test the relationship between phiclust and the tARI in experimentally measured data, we used an scRNA-seq data set of peripheral blood mononuclear cells (PBMCs) [22]. We chose two very distinct cell types and created new clusters as weighted, linear combinations of expression profiles from the two cell types. This approach allowed us to precisely control the difference between the newly created clusters, while maintaining the experimentally observed noise structure (Additional file 1: Fig. S3e). Also for this data, phiclust strongly correlates with the tARI (Fig. 1d). As an alternative to the tARI, we also calculated the theoretically achievable silhouette coefficient [8] (tSIL), which considers the distances between the best possible clusters (Additional file 1: Fig. S4 a-c). For a large range of simulation parameters, the tSIL has a smaller dynamic range than the tARI, which makes it less useful overall for assessing clusterability. In contrast to phiclust, ROGUE [9] does not show collinearity with the tARI (Additional file 1: Fig. S4d). Therefore, ROGUE seems to implement a notion of clusterability that is distinct from our point of view.

### Confounder regression removes unwanted variability

To further characterize the performance of phiclust on experimental data sets with known ground truth, we used a measurement of purified RNA from 3 cell types, mixed at different ratios [23] (Fig. 1e). We noticed a significant correlation between the amount of input RNA and the entries of the first singular vector of individual clusters (Fig. 1f). This might be explained by lowly expressed genes not being well-represented in the low-input libraries and the resulting differences in the expression profiles. In any case, the amount of input RNA seemed to be a confounding factor that could lead to high values of phiclust, even in the absence of meaningful subclusters. Correspondingly, we found a correlation between the singular vector entries and the number of total counts, despite normalization of the data (Fig. 1g). This is consistent with the finding that total counts are a confounding factor in scRNA-seq data that cannot be eliminated by normalization using one single scaling factor per cell [22, 24]. Different groups of genes scale differently with the total counts per cell. Therefore, a correlation with the total counts remains even after normalization.

More generally, there are various experimental and biological factors that drive artefactual or irrelevant variability in single-cell RNA-seq data [22, 25]. We therefore introduced a regression step that removes the influence of any nuisance variables, such as the number of total counts per cell, ribosomal gene expression, mitochondrial gene expression, or cell cycle phase (see also Additional file 2). More specifically, we first regress the entries of a singular vector on one or multiple confounders. The fraction of variance explained by all confounder is then given by the adjusted R^2^ (coefficient of determination) of the linear regression. Since the squared singular values can also be interpreted as the amount of variance explained, we correct them by multiplying with 1 − the adjusted *R*^2^ found in the confounder regression. (See Table S2 for a list of the uncorrected and corrected singular values for both simulated and experimental expression matrices.) The corrected singular values are then used to calculate phiclust.

Interestingly, the relative influence of the confounders considered in this study varied substantially between data sets (Additional file 1: Fig. S5a). For example, cell stress is a relevant confounder only in the kidney data set. This is likely related to the cell dissociation procedure, which is necessarily more aggressive for kidney tissue, compared to the other samples: bone marrow mononuclear cells (BMNCs) and purified RNA, extracted from cell cultures. Total counts and ribosomal gene expression explain most of the artefactual variance in BMNCs. This might be explained by high variability in the metabolic state of the cells. In Table S2 we list the *R*^2^ values of each considered confounder for each cluster. For real scRNA-seq data sets, confounder regression can lead to a significant reduction of phiclust (Additional file 1: Fig. S5b, see Table S2 for the numerical values.) It is therefore an important part of the method.

Confounder regression can also help to analyze data sets that are not made up of regular clusters but contain irregularly shaped continua of gene expression. For example, in developmental and stem cell biology, we commonly observe differentiation paths, which are large clusters with gradually changing expression profiles. Uncorrected phiclust values are high for such paths, which suggests meaningful subpopulations (S5c,d). Depending on the biological question, it might in fact be desirable to cluster differentiation paths, for example, to separate a stem cell state from a differentiated cell type. For other applications, it could be preferable to treat a differentiation path as one cluster. In that case, we can use pseudotime approaches [26] to infer a temporal order of the gene expression profiles and use the inferred pseudotime in the confounder regression. If all observed variability is explained by developmental dynamics, phiclust is reduced to 0 and thus no sub-clustering is suggested (S5c,d).

### Phiclust has high sensitivity for the detection of sub-structure

After correction for unwanted variability, we compared the performance of phiclust with other clusterability measures in the RNA mixture data set (Fig. 1e). Phiclust successfully indicated the presence or absence of subclusters for all tested combinations of the 7 original mixtures (Additional file 1: Fig. S6). By contrast, ROGUE only indicated the presence of substructure when the merged clusters were very clearly distinguishable (Additional file 1: Fig. S6 b,c). The silhouette coefficient was qualitatively similar to phiclust but its dynamic range was much smaller (Additional file 1: Fig. S6, middle row). This might become critical in the case of highly similar phenotypes, which is precisely where phiclust might have an advantage. An example for this can be seen in Additional file 1: Fig. S6b: the silhouette coefficients in the pure cluster are very similar to the merged clusters (which were composed of two original clusters). To compare phiclust with the silhouette coefficient in more detail, we carried out additional simulations (Additional file 1: Fig. S7). First, we simulated 3 clusters and subsequently merged two of them. While phiclust clearly distinguished the merged cluster from the pure cluster, the silhouette coefficients were similar for both. Increasing the fraction of genes that are differentially expressed between the merged cluster increased the difference in silhouette coefficient, but only gradually (Additional file 1: Fig. S7b). By contrast, phiclust jumped to values close to 1 for the merged cluster for very small fractions of differentially expressed genes (around 0.03). It is therefore the more sensitive measure. The silhouette coefficient strongly depends on the number of principal components used in dimensionality reduction (Additional file 1: Fig. S7c), as well as the metric for distances between expression profiles (Additional file 1: Fig. S7d). Phiclust does not depend on such user-defined parameters. Most importantly, the silhouette coefficient cannot answer the question, whether an identified cluster contains meaningful substructure, as it requires partitioning into at least 2 sub-clusters. We simulated a cluster without any substructure and all variability was purely random (Additional file 1: Fig. S7e). The silhouette coefficient was maximal for a k-means clustering with *k* = 2, which might prompt a user to conclude (wrongly) that there are 2 sub-clusters present. Phiclust, which does not require any further partitioning of the cluster, was 0, indicating correctly that the observed variability was consistent with random noise. All in all, these comparisons indicate that phiclust is a sensitive measure, which detects differences between highly similar phenotypes.

### Genes responsible for the detected substructure can be identified

In full analogy to the reasoning outlined so far, our approach can also be used to characterize variability in gene space, for which we defined the conjugate measure g-phiclust (see Additional file 2 for the derivation). Above, we considered only the right singular vectors, where each entry corresponds to a cell in the data set. We therefore also call them “cell-singular vectors.” In the simplest case of well separated clusters, entries in the cell singular vectors indicate the membership of a cell in a cluster or a group of clusters. For the left singular vectors, each entry corresponds to a gene. Therefore, we also call them “gene-singular vector.” The squared cosine of the angle between the leading gene-singular vector in the measured gene expression matrix and the corresponding gene-singular vector of the noise-free signal matrix is g-phiclust. As for phiclust, data sets with higher signal-to-noise ratios are characterized by higher values of g-phiclust (Additional file 1: Fig. S8a). “Signal” and “noise” are defined exactly as above: “noise” is a random matrix and the “signal” is a low-rank matrix consisting of noise-free expression profiles, where the strength of the signal (or difference between the clusters) corresponds to the magnitude of the non-zero singular values. A g-phiclust close to 0 would indicate that all observed differential gene expression can be explained by random noise. Larger values of g-phiclust indicate less overlap of the gene expression profiles between phenotypes. We therefore expect to find a bigger number of significantly differentially expressed (DE) genes and/or larger fold changes between phenotypes. We confirmed by simulations that genes with larger absolute entries in a gene-singular vector contribute more to the differences between the clusters separated along the corresponding cell-singular vector (Additional file 1: Fig. S8b-d): For example, if two clusters (A and B) are separated along a cell-singular vector and cells in cluster A are characterized by positive entries, the genes with large positive entries in the corresponding gene-singular vector will be mostly expressed in cluster A. We call these “variance driving” genes. Our approach thus not only identifies relevant substructure in a cell cluster but can also reveal the genes responsible for it. In contrast to differential expression tests, the variance driving genes can be obtained before clustering and might help the user interpret the observed variability and make an informed decision on whether it is useful to sub-cluster the data. If the variance driving genes have enriched biological features (such as being involved in the same signaling pathway or cellular function), we can take that as additional evidence for biologically meaningful sub-population.

### Application of phiclust to a BMNC data set drives the discovery of biologically meaningful sub-clusters

The most important application of phiclust, in our opinion, is to prioritize clusters for further sub-clustering and follow-up studies. For a complex tissue with dozens of clusters, it is not feasible to sub-cluster all of them and try to validate all resulting subpopulations. This is particularly inefficient, if many subclusters are in fact just driven by random noise. High values of phiclust nominate those clusters that likely have deterministic structure and are therefore worthwhile to be scrutinized experimentally in more detail.

**Fig. 2:**
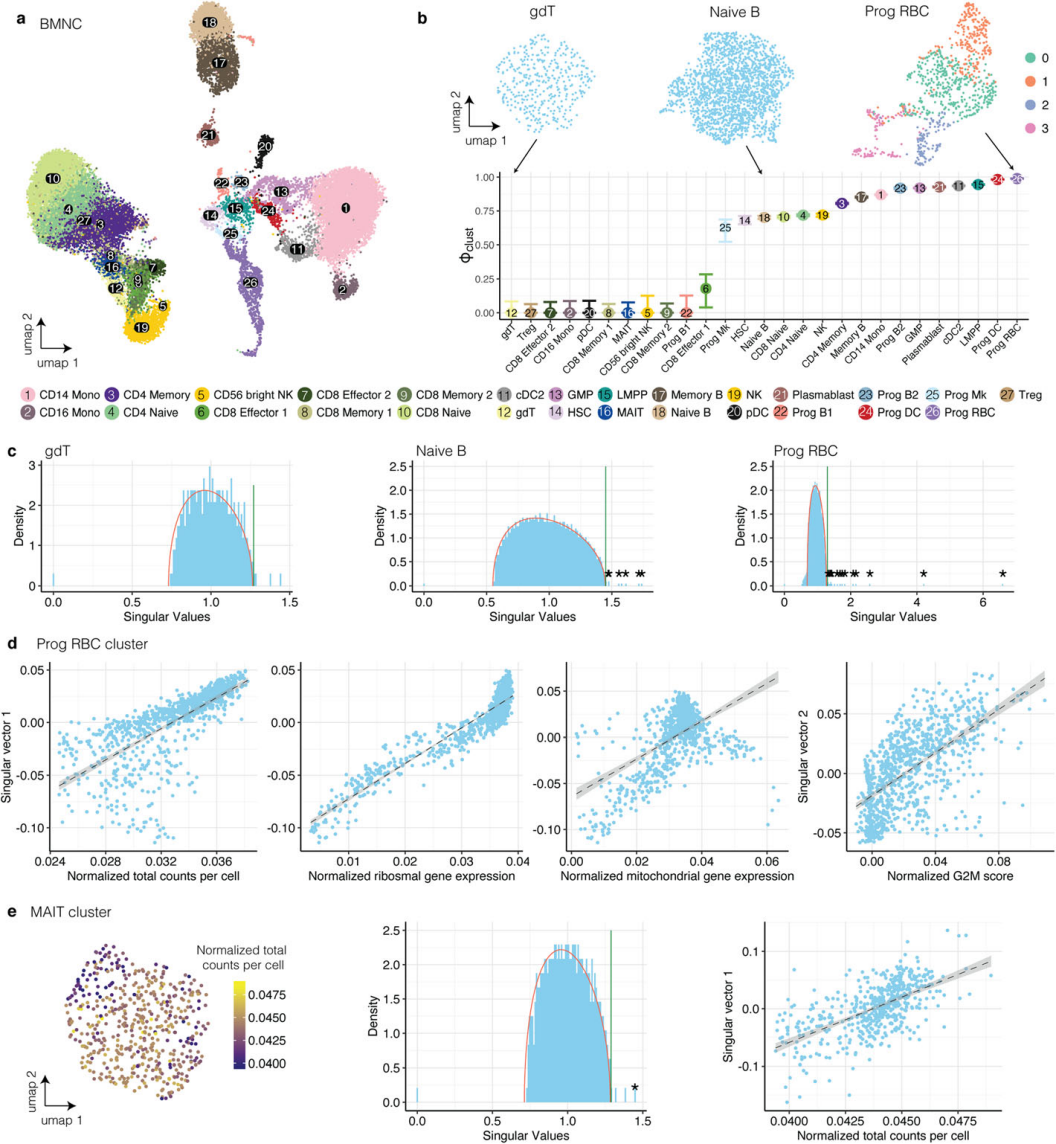
Application of phiclust to a BMNC data set drives the discovery of biologically meaningful sub-clusters. **a** UMAP of BMNC data set. **b** Phiclust for the BMNC data set. Error bars indicate the uncertainty obtained by resampling the noise. Inset: UMAP of clusters with low, intermediate, and high values of phiclust. **c** Singular value distribution, MP distribution (red line), and TW threshold (green line) of clusters with low, intermediate, and high values of phiclust. Significant singular values are highlighted with asterisks. In the gdT cluster, the singular vectors corresponding to the outlying singular values had normal distributed entries and were thus not significant. **d** First three graphs: first singular vector of the red blood cell progenitor cluster in the BMNC data set versus normalized total counts per cell, normalized expression of ribosomal genes, and normalized expression of mitochondrial genes. Rightmost graph: second singular vector versus normalized G2M score. The dashed line indicates the linear regression and the grey area indicates the standard deviation. **e** Left: UMAP of the MAIT cell cluster in BMNC data set. The color indicates the normalized total counts per cell. Middle: singular value distribution, MP distribution (red line), and TW threshold (green line) for the MAIT cell cluster. The only significant singular value is indicated by an asterisk. Right: normalized total counts per cell versus the singular vector associated with the significant singular value (here: first singular vector) in the MAIT cluster

To demonstrate the application of phiclust and g-phiclust, we analyzed scRNA-seq measurements of complex tissues. In a data set of bone marrow mononuclear cells (BMNCs) [27], we calculated phiclust for the clusters reported by the authors (Fig. 2a,b). For all clusters, except the red blood cell (RBC) progenitor cluster, the bulk of the singular value distribution was well-described by the MP distribution. (In the RBC progenitors, we found several singular values below the lower limit of the MP distribution. These outliers did not influence the further analysis since we are only interested in singular vectors above the upper limit.) The first cell-singular vectors of all clusters were significantly correlated with several confounding factors (see Fig. 2d for RBC progenitors and Fig. 2e for MAIT cells). After correction for these confounding factors, phiclust corresponded well with a visual inspection of the cluster UMAPs (Fig. 2b): Where obvious clusters were present, phiclust was highest, while homogeneous, structure-less clusters resulted in a phiclust of 0. Reassuringly, many progenitor cell types received a high phiclust (indicating possible substructure) in agreement with the known higher variability in these cell types. Ranking existing clusters by g-phiclust resulted in a very similar order (Additional file 1: Fig. S9a).

To confirm the presence of relevant substructure, we subclustered the two original clusters with the highest phiclust (Additional file 1: Fig. S9 b-e). In the RBC progenitors, we identified 4 subsets that correspond to different stages of differentiation, ranging from erythroid precursors to highly differentiated RBCs, as identified previously [28]. In the dendritic cell (DC) progenitor cluster, two subclusters were identified, which correspond to precursors of classical or plasmacytoid DCs, respectively [29]. For both examples, the variance-driving genes found in the gene-singular vectors were localized to their corresponding clusters (Additional file 1: Fig. S9 c,d) and overlapped strongly with differentially expressed genes found after subclustering (see Table S3).

### Phiclust reveals subpopulations in a fetal human kidney data set that can be confirmed experimentally

**Fig. 3:**
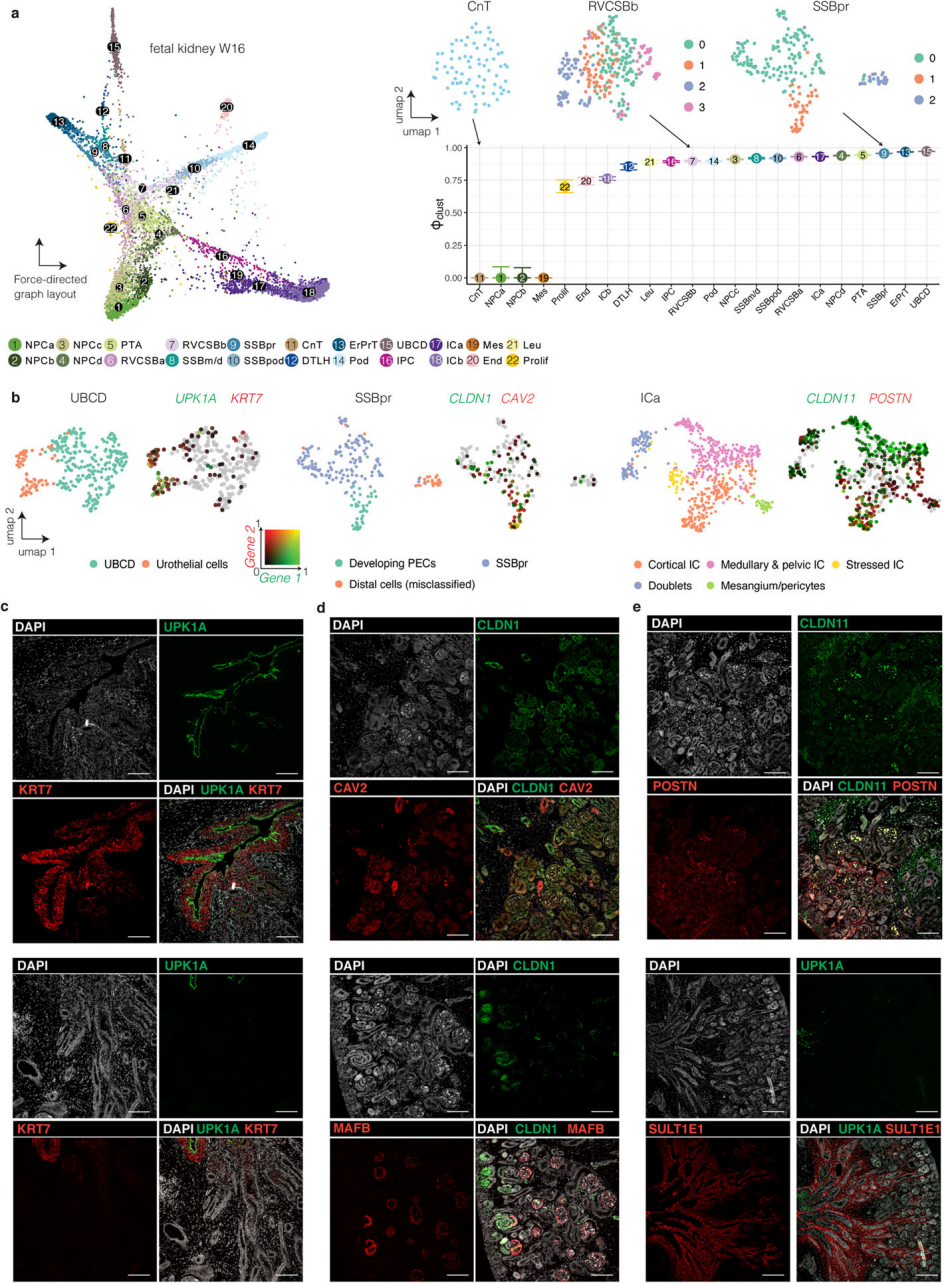
Phiclust reveals subpopulations in a human fetal kidney data set that can be confirmed experimentally. **a** Force-directed graph layout and phiclust for the fetal kidney data set. Error bars indicate the uncertainty obtained by resampling the noise. Inset: UMAP of clusters with low, intermediate, and high values of phiclust. **b** UMAPs of the UBCD, SSBpr, and ICa clusters. Left: colors indicate sub-clusters. Right: colors indicate the log-normalized gene expression of the two indicated genes. One gene follows the red color spectrum, the other gene the green color spectrum. Absence of color indicates low expression in both genes, yellow indicates co-expression of both genes. **c**–**e** Immunostainings of week 15 fetal kidney sections. **c** UPK1A and KRT7 are expressed in the urothelial cells of the developing ureter (upper panel) and absent from the tubules in the adjacent inner medulla (lower panel). **d** PECs express CLDN1 and CAV2 (upper panel), as well as CLDN1 at the capillary loop stage and later stages (lower panel). MAFB staining is found in podocytes and their precursors in the SSB (lower panel). **e** CLDN11 and POSTN are expressed in interstitial cells visualized by immunostaining (upper panel). SULT1E1 is expressed in the interstitial cells surrounding the ureter (marked by UPK1A) and the tubule in the inner medulla (lower panel). Scale bars: 100 μm

As a second example of our approach, we analyzed a fetal human kidney data set we published previously [30]. In our original analysis, we were forced to merge several clusters, since we were unsure if the observed variability was just noise. We hence wanted to use phiclust to find previously overlooked subpopulations. As for BMNCs, phiclust corresponded well with a qualitative assessment of cluster variability (Fig. 3a): Clusters with visible sub-clusters had the highest values of phiclust. Ordering the clusters by g-phiclust resulted in a similar ranking as phiclust (Additional file 1: Fig. S10a).

Subclustering of the cluster with the highest phiclust, ureteric bud/collecting duct (UBCD), revealed a subset of cells with markers of urothelial cells (*UPK1A*, *KRT7*) (Fig. 3b, Additional file 1: Fig. S10b-e). Immunostaining of these two genes, together with a marker of the collecting system (CDH1), in week 15 fetal human kidney sections confirmed the presence of the urothelial subcluster (Fig. 3c, Additional file 1: Fig. S11a).

Another cell type we did not find in our original analysis, are the parietal epithelial cells (PECs). They could now be identified within the SSBpr cluster (S-shaped body proximal precursor cells) (Fig. 3b, Additional file 1: Fig. S10b-e). To reveal these cells in situ, we stained for AKAP12 and CAV2, which were among the top differentially expressed genes in this subcluster (Table S4), together with CLDN1, a known marker of PECs, and MAFB, a marker of the neighboring podocytes (Fig. 3d, Additional file 1: Fig. S11b). Next to the PECs and proximal tubule precursor cells, SSBpr also contained a few cells that were misclassified in the original analysis, indicating the additional usefulness of phiclust as a means to identify clustering errors.

Further analysis of a cluster of interstitial cells (ICa) revealed multiple subpopulations (Fig. 3b, Additional file 1: Fig. S10b-e). Immunostaining showed that a POSTN-positive population is found mostly in the cortex, often surrounding blood vessels, whereas a SULT1E1-positive population is located in the inner medulla and papilla, often surrounding tubules (Fig. 3e, Additional file 1: Fig. S11c). CLDN11, another gene identified by analysis of the gene-singular vectors (Additional file 1: Fig. S10b-e), was found mostly in the medulla, but also in the outermost cortex. A more detailed, biological interpretation of the results can be found in Additional file 3.

## Discussion

Here, we presented phiclust, a clusterability measure that can help detect subtly different phenotypes in scRNA-seq data. Universal properties of the underlying theory make it possible to apply phiclust to arbitrary noise distributions, and the noise can be additive or multiplicative. Empirically, we find that the bulk of the singular value distribution of measured expression matrices is well-approximated by the MP distribution. This supports the assumption that the noise is generated by independent and identically distributed random processes.

While most of the technical and biological noise can likely be considered random, there are also known systematic errors and unwanted, confounding factors (such as the efficiency of RNA recovery, cell cycle phase etc.) Therefore, regressing out uninformative, deterministic factors, is an important part of the method.

The approach underlying phiclust also allows us to identify the genes that are most relevant for the biological interpretation of the observed variability. We found these genes to overlap strongly with differentially expressed genes identified after sub-clustering. The g-phiclust measure, a conjugate to phiclust, quantifies how distinguishable the expression profiles of different phenotypes are in the presence of noise.

The most important application of phiclust is the nomination of clusters for sub-clustering and subsequent experimental validation. All clusters that were nominated in the fetal kidney data set turned out to have subpopulations that could be validated by experiments: rare urothelial cells, which differ from nearby clusters in only a few genes; PECs and subtypes of interstitial cells, which had distinct spatial distributions.

There are several other methods that attempt to detect the presence of meaningful information in single-cell RNA-seq data. Below, we will compare phiclust to some of the most popular examples: the silhouette coefficient, ROGUE, robust PCA, the dip test, and ZINB-WaVE.

The silhouette coefficient is a popular tool to assess clustering quality. In contrast to phiclust, this coefficient requires a (sub-)clustering, and it cannot be used to decide, whether a cluster contains meaningful variability and should be sub-clustered further. As demonstrated, using the silhouette coefficient can lead to over-clustering of random noise as well as missing the presence of subtly different phenotypes. Likewise, phiclust appeared to be more sensitive than ROGUE, an entropy-based clusterability measure. Both ROGUE and the silhouette coefficient do not scale linearly with the tARI, which we introduced as an upper limit to the achievable agreement of an empirical clustering with the ground truth.

Robust PCA [31, 32] decomposes a measured expression matrix into a sparse component and a low-rank component. Under the assumption that noise is sparse, the sparse component is identified with random noise. In our opinion, there is no reason to assume that the noise in scRNA-seq data is sparse or sparser than the measured expression matrix itself. Likely, every non-zero gene expression measurement was corrupted by noise. Additionally, the remaining low-rank component cannot be identified as the noise-free signal. It is fundamentally impossible to reconstruct the noise-free signal from the measured expression because the noise is created by a random process. The low-rank component is therefore only a (noisy) approximation of the noise-free signal. Given the fundamental limit to signal reconstruction, the best thing we can do is quantify the closeness between signal and measured expression, as implemented by phiclust. In robust PCA, the low-rank matrix is often further subjected to dimensionality reduction, where it is difficult to determine the correct number of dimensions. phiclust does not require any dimensionality reduction and uses all available data.

The dip test [7], a method aimed at detecting the presence of clusters, tests whether there are multiple modes in the data. It can be applied directly to the distribution of distances between expression profiles or a low-dimensional representation of the data, such as principal component scores. The dip test will miss relevant variability, if it does not manifest itself as separate modes, which can easily occur, for example in the case of differentiation paths. It also just provides a binary result (modes present or not), whereas phiclust is a continuous measure and does not require the presence of modes.

ZINB-WaVE [24] performs dimensionality reduction based on a zero-inflated negative binomial distribution and is similar to principal component analysis, if no additional covariates are added to the model. ZINB-WaVE acknowledges the fact that principal components are prone to correlate with nuisance parameters, even after normalization. The problem is circumvented by adding such parameters as covariates to the model, which is similar to the confounder regression used for phiclust. However, the user has to decide the number of dimensions to use and currently there is no principled way to determine the optimal number. phiclust does not have any such adjustable parameters.

## Conclusion

We hope that this manuscript will bring renewed awareness to random noise as a factor that imposes hard limits on clustering and identification of differentially expressed genes. We hope that quantitative measures of clusterability, such as phiclust, can play an important role in making single-cell RNA-seq analysis more reproducible and robust.

## Methods

### Preprocessing

Before applying the method to simulated or measured single-cell RNA-seq data sets, several preprocessing steps are necessary. The raw counts are first normalized and log-transformed. Next, the expression matrix is standardized, first gene-wise, then cell-wise. These steps assure the proper agreement of the bulk of the singular value distribution with the MP distribution (Additional file 1: Fig. S2). See also Additional file 2, Section 3.1.

### Phiclust

To derive phiclust, we assume that the expression matrix $\overset{\sim }{X}$ measured by scRNA-seq, can be written as the sum of a random matrix *X*, which contains random biological variability and technical noise, and a signal matrix *P*, which contains the unobserved expression profiles of each cell:

$$ \tilde{X}=X+P $$

Note that in this decomposition, cells that belong to the same cell type (or phenotype) have identical expression profiles in the signal matrix *P*. Below, we will show that multiplicative noise can be treated analogously.

We apply SVD to obtain the singular values, as well as the right and left singular vectors of $\overset{\sim }{X}$. The left singular vectors span gene-space and the right singular vectors span cell-space. Hence, we call them gene-singular vectors and cell-singular vectors, respectively. If we use the term “singular vector” it is implied to mean cell-singular vector.

Considering the signal matrix *P* a perturbation to the random matrix *X*, we can apply results from both random matrix theory and low-rank perturbation theory. Random matrix theory [33, 34] predicts that the singular value distribution of *X* is a Marchenko-Pastur (MP) distribution [17, 18, 35], which coincides with the bulk of the singular value distribution [11–13] of $\overset{\sim }{X\ }$. The singular values of $\overset{\sim }{X\ }$above the values predicted by the MP distribution characterize the signal matrix *P*. Since the agreement with the MP distribution holds strictly only for infinite matrices, we use two additional concepts to identify relevant singular values exceeding the range defined by the MP distribution. The Tracy-Widom [15, 35] (TW) distribution describes the probability of a singular value to exceed the MP distribution, if the matrix is finite. Additionally, since singular vectors of a random matrix are normally distributed, relevant singular vectors have to be significantly different from normal [13]. To test for normality, we used the Shapiro-Wilk test.

We apply low-rank perturbation theory [16] to calculate the singular values (*θ*_*i*_) of *P* from the outlying singular values (*γ*_*i*_) of the measured expression matrix $\overset{\sim }{X\ }$:

$$ {\theta}_i\left({\gamma}_i\right)=\sqrt{\frac{2c}{{\gamma_i}^2-\left(c+1\right)-\sqrt{{\left({\gamma_i}^2-\left(c+1\right)\right)}^2-4c}}} $$

where *c* is the cell-to-gene ratio, i.e., the total number of cells divided by the total number of genes.

The values of *θ*_*i*_ are then used to obtain the angles *α*_*i*_ between the singular vectors of $\overset{\sim }{X}$ and *P*. These angles are conveniently expressed in terms of their squared cosine as

$$ {\phi}_i=\mathit{\cos}{\left({\alpha}_i\right)}^2=1-\frac{c\left(1+{\theta}_i^2\right)}{\theta_i^2\left({\theta}_i^2+c\right)} $$

The leading singular vector of the measured expression matrix, which has the largest singular value, has the smallest angle to its unperturbed counterpart. The squared cosine of this smallest angle is then used as a measure of clusterability:

$$ {\phi}_{clust}=\mathit{\cos}{\left( mi{n}_i{\alpha}_i\right)}^2= ma{x}_i\mathit{\cos}{\left({\alpha}_i\right)}^2= ma{x}_i{\phi}_i,{\phi}_i\in \left[0,\pi /2\right]. $$

For a detailed derivation of phiclust, see Additional File 2, Sections 2.1–2.4.

### Uncertainty of phiclust

The uncertainties for the values phiclust are estimated using a sampling approach. The basic idea is to approximate the signal matrix *P* and add new realizations of the noise matrix by sampling from a random distribution. The uncertainty is then obtained from the values phiclust calculated for this ensemble of sampled matrices. First, we decompose a simulated or measured expression matrix $\overset{\sim }{X}$ into a noise matrix *X*^r^ and a matrix *X*^s^ that contains deterministic structure. *X*^s^ is constructed from the relevant singular vectors, which were identified as described in the previous section. Note that *X*^s^ contains noise and is thus different from the signal matrix *P*. To create an approximation *P*^s^ of the signal matrix *P*, we replace the singular values *γ*_*i*_ used in the construction of *X*^s^ with the singular values *θ*_*i*_ of *P*, calculated using low-rank perturbation theory as shown in the previous section. The entries of the noise matrix *X*^r^ have a mean of 0 and a standard deviation of 1, as a result of preprocessing. Since the results of RMT are valid irrespective of the particular noise distribution, we can create additional realizations of the noise matrix by sampling from a normal distribution with mean 0 and standard deviation 1. By adding sampled noise matrices to the approximated signal matrix *P*^s^, we can create an ensemble of matrices with the same singular value spectrum as the original measured expression matrix but different realizations of the noise. The uncertainty for positive and negative deviations from the mean is then calculated as the standard deviation for at least 50 sampled matrices. See Additional file 2, Section 2.4.3 for a detailed description.

### Test for deviation from the MP distribution

To validate the use of the MP distribution, we test whether the bulk of the measured singular value distribution deviates significantly. Singular values are considered to be part of the bulk, if they are located below the MP upper bound and not associated with the transcriptome mode. We sample 1000 values from the MP distribution using the RMTstat R package (V 0.3) and subsequently test for similarity with the Kolmogorov-Smirnov test [36]. The resulting *p* values are adjusted for multiple hypothesis testing with the Benjamini-Hochberg procedure [37].

### Confounder regression

scRNA-seq data contains various confounding factors that drive uninformative variability. These either emerge from technical issues (such as the varying efficiency of transcript recovery, which cannot be fully eliminated by normalization) or biological factors (such as cell cycle phase, metabolic state, or stress). To account for these factors, a regression step, inspired by current gene expression normalization methods [22, 25], is included. We perform a linear regression by using each relevant singular vector as a response variable and the confounding factors as covariates. This is a valid approach because the singular vectors of the measured expression matrix contain normal distributed noise. The amount of variance explained by the nuisance parameters is then given by the value of the adjusted *R*^2^ (coefficient of determination) of this linear regression. To relate the regression result to the singular values, we consider the squared singular values (= eigenvalues) which correspond to the variance explained by the corresponding singular vectors/eigenvectors. Squared singular values are corrected by multiplication with (1 − adjusted *R*^2^) to retrieve the fraction of variance not explained by nuisance parameters. The square root of the result is the corrected singular value See also Additional file 2, Section 3.2. For Additional file 1: Fig. S5a, each nuisance parameter was individually regressed on, to compare the influence of each factor.

### Multiplicative noise

To model multiplicative noise, we use a rectangular random noise matrix *X* with the same dimensions as the measured expression matrix $\overset{\sim }{X}$ and a square signal matrix *P* whose number of rows or columns is equal to the number of measured genes. The measured expression matrix $\overset{\sim }{X}$is then modeled as:

$$ \overset{\sim }{X}={\left(I+P\right)}^{\frac{1}{2}}\ X, $$

where *I* denotes the identity matrix. Importantly, the bulk of the singular vector distribution of the measured expression matrix $\overset{\sim }{X}$ still follows the MP distribution in this model. The singular values of the signal matrix *P* are calculated from the outlying singular values of $\overset{\sim }{X}$ by:

$$ {\uptheta}_{\mathrm{i}}=\frac{2c}{\uplambda_{\mathrm{i}}-c-1-\sqrt{\left({\uplambda}_{\mathrm{i}}-a\right)\left({\uplambda}_{\mathrm{i}}-b\right)}} $$

with $\mathrm{a},\mathrm{b}={\left(1\pm \sqrt{c}\right)}^2$. The squared cosine of the angles between the corresponding singular vectors of the measured expression matrix and the signal matrix are then calculated as:

$$ {\upphi}_i^{mult}=\frac{1}{\uptheta_{\mathrm{i}}}\frac{{\uptheta_{\mathrm{i}}}^2-c}{\uptheta_{\mathrm{i}\left(c+1\right)}+2c} $$

As before, the largest of these values is taken to be the clusterablity measure. More information on multiplicative perturbation can be found in [38].

### Theoretically achievable clustering quality

To construct a Bayes classifier [21], which achieves the minimal error rate, we need to know the ground truth clustering. Hence, we used data simulated with Splatter [19], containing two clusters. For each ground truth cluster, we fit a multidimensional Gaussian to the corresponding entries of the singular vectors (see Additional file 1: Fig. S3a). We only consider singular vectors with singular values larger than predicted by the MP distribution. For the fit, we use the mclust [39] R package (V 5.4.6). We then construct a classifier by assigning a cell to the cluster for which it has the highest value of the fitted Gaussian distribution. This classifier is thus approximately a Bayes classifier (for a true Bayes classifier, we would need to know the exact distributions of the singular vector entries). The ARI [20] calculated based on this classification is thus approximately the best theoretically achievable ARI (tARI).

We also tested the silhouette coefficient [8] as a potential alternative to the ARI for quantifying our notion of clusterability. The silhouette coefficient was calculated on the first singular vector using Euclidean distances. In Additional file 1: Fig. S4, the silhouette coefficient averaged over all cells is reported. The theoretically achievable silhouette coefficient tSIL is defined as the silhouette coefficient of the Bayes classification described in the previous paragraph. The calculation of tARI and tSIL is described in more detail in Additional file 2, section 2.5.

### Clustering methods

For the validation of the tARI and tSIL, several clustering methods were used on simulated data with two clusters. Seurat clustering [1] was performed with the *Seurat R package* with 10 principal components (PCs) and 20 nearest neighbors. Three different resolution parameters were used: 0.1, 0.6, and 1.6. Scanpy clustering [2] was performed with the *scanpy python package* with 10 PCs and 20 nearest neighbors. Three different resolution parameters were used: 0.1, 0.6, and 1.6. Hierarchical clustering [4] was performed on the first 10 PCs and Euclidean distances. The hierarchical tree was built with the Ward linkage and the tree was cut at a height where 2 clusters could be identified. K-means [3] was performed on the first 10 PCs using Euclidean distances and two centers. TSCAN [40] was calculated on the first 10 PCs. In Additional file 1: Fig. S7, k-means clustering was performed on the first 3 principal components and using Euclidean distances.

### Clusterability measures

ROGUE [9] is an entropy-based clusterability measure. A null model is defined under the assumption of Gamma-Poisson distributed gene expression and its differential entropy is then compared to the actual differential entropy of the gene expression profile. For the RNA-mix data set, ROGUE (V 1.0) was used with 1 sample (see Fig S6), “UMI” platform, and a span of 0.6. For the simulated data sets, ROGUE was used with *k* = 10 (Additional file 1: Fig. S4 d). The silhouette coefficient was calculated with the cluster R package (V 2.1.0) using Euclidean distances in the space of the relevant singular vectors. The reported values for the silhouette coefficients are average values per cluster. The confidence intervals given in Additional file 1: Fig. S6 and S7 are standard deviations of its values per cluster.

### Variance driving genes

Genes that drive the variance in the significant singular vectors can be used to explore the biological information in the sub-structures. Genes with large positive or negative entries in a gene-singular vector are localized in cells with high positive or negative entries in the corresponding cell-singular vector. It is also possible to assess the signal-to-noise ratio for the genes by calculating the squared cosine of the angle between the gene singular vectors of the measured expression matrix $\overset{\sim }{X}$ and the gene singular vectors of the signal matrix *P*, given by^15^

$$ {\phi}_i^g=\mathit{\cos}{\left({\overset{\sim }{\alpha}}_i\right)}^2=1-\frac{\left(c+{\theta}_i^2\right)}{\theta_i^2\left({\theta}_i^2+1\right)}, $$

where *c* is the cell-to-gene ratio. The largest of the ${\phi}_i^g$is then called ${\phi}_{clust}^g$, the gene phiclust (g-phiclust). See Additional file 2, section 2.4 for a more detailed discussion.

### Data sets

The simulated data sets in Additional file 1: Fig. S1 comprised 201 cells and 350 genes. The random noise matrix was sampled from a normal distribution with mean 0 and variance 1 in panels a and b, or from a Poisson distribution with parameter 1 in panels c and d. The rank 1 signal matrix was constructed from one cell-singular vector and one gene-singular vector. The cell-singular vector consisted of 67 entries equal to $1/\sqrt{N_{cell}}$ and all other entries equal to $-1/\sqrt{N_{cell}}$ , where *N*_cell_ is the number of cells. The gene-singular vector consisted of 200 entries equal to $1/\sqrt{N_{gene}}$ and the rest equal to $-1/\sqrt{N_{gene}}$ , where *N*_gene_ is the number of genes. The signal matrix was then created by matrix multiplication of the gene-singular vector and the transposed cell-singular vector times the singular value θ (θ = 2 in a,c and *θ* = 5 in b,d). In Additional file 1: Fig. S2f, g a rank 1 signal matrix was created similarly as described above. The cell-singular vector with a number of entries matching the number of cells in the cluster was constructed as before. The gene-singular vector was drawn from a normal distribution and subsequently normalized to unit length. The rank 1 signal matrix was then added to the preprocessed expression matrix of the indicated cluster.

The remaining simulated data sets were produced with the *splatter* [19] *R package (V 1.10.1)*. The parameters used for the simulation are shown in Table S1. For Fig. 1c,d, Additional file 1: Fig. S3b-e, Additional file 1: Fig. S4, and Additional file 1: Fig. S8a, the simulations for each parameter were performed 50 times, each with a different seed. The results were averaged over the 50 runs. Confounder regression was performed for the total number of transcripts per cell.

PBMC data [22] was downloaded from the 10x genomics website (https://cf.10xgenomics.com/samples/cell/pbmc3k/pbmc3k_filtered_gene_bc_matrices.tar.gz). For the calculation of the tARI, clustering with Scanpy, TSCAN, *k*-means, and hierarchical clustering, preprocessing was performed with the *scanpy python package (V 1.4.6)* following the provided pipeline (https://scanpy-tutorials.readthedocs.io/en/latest/pbmc3k.html) for the filtering of cells and genes, normalization, and log-transformation as well as cluster annotation. For the clustering with Seurat, the provided Seurat pipeline was used (https://satijalab.org/seurat/archive/v3.2/pbmc3k_tutorial.html) for preprocessing, such as cell and gene filtering, normalization, log-transformation, and cluster annotation using the Seurat R package (*V 3.1.5*). CD8 T cells and B cells were extracted from the data, and each cluster was standardized gene-wise and cell-wise before the calculation of the SVD. To remove any sub-structure in these clusters and before the reconstruction of the matrices from the SVD, singular values above the MP distribution were moved into the bulk, and the transcriptome mode (i.e., the singular vector that would have the largest singular value without normalization, see Additional file 2) was moved above the MP distribution. Then, two synthetic clusters containing 150 cells each were created from the cleaned-up original clusters. For cluster 1, a weighted average of a randomly picked B cell with expression profile *c*_*B*_ and a randomly picked CD8 T cell with expression profile *c*_*CD*8 *T*_ was calculated according to: *c*_1_ = *α* ∙ *c*_*B*_ + (1 − *α*) ∙ *c*_*CD*8 *T*_. For cluster 2, the weights were flipped: *c*_2_ = (1 − *α*) ∙ *c*_*B*_ + *α* ∙ *c*_*CD*8 *T*_. *α* was chosen in a range from 0 to 1. *α* close to 0.5 produced highly similar clusters, while *α* close to 0 or 1 produced maximally different clusters (see Fig S3e). For each value of *α*, the procedure was repeated 50 times, each with a different seed for selecting 300 cells per cell type, and the results were averaged.

RNA-mix data [23] was downloaded from the provided GitHub page. The data were normalized with the R scran package (V 1.14.6) and then log-transformed. Confounder regression was performed for the total number of transcripts, average mitochondrial gene expression, and average ribosomal gene expression. Two different merged clusters were created from the provided RNA mixtures as shown in Additional file 1: Fig. S6.

The bone marrow mononuclear cell data set (BMNC) [27] was downloaded from the *R package SeuratData (bmcite, V 0.2.1)*. Normalization and calculation of the G2M score [41] were performed with the *Seurat R package (V 3.1.5*). Confounder regression was performed for the log-transformed total number of transcripts, cell cycle score, and average expression of: mitochondrial genes and ribosomal genes (list obtained from the HGNC website).

For the fetal kidney data set [30], the same preprocessing and normalization was used as reported previously (scran R package [42]). The data was then log-transformed and the G2M score was calculated with the *Seurat R package*. Confounder regression was performed for the log-transformed total number of transcripts, G2M scores, and the average expression of: mitochondrial genes, ribosomal genes, and stress-related genes [43].

### Embedding

Uniform Manifold Approximation and Projections [44] (UMAPs) for individual clusters were calculated with the R package umap (V 0.2.7.0) on the first 10 PCs, 20 nearest neighbors, min_dist = 0.3, and Euclidean distances. The umap for BMNC data was calculated with the *Seurat R package* using 2000 highly variable genes (hvg), *d* = 50, *k* = 50, min.dist = 0.6, and metric = cosine. For the fetal kidney data set, a force-directed graph layout was calculated using the *scanpy* python package. The graph was constructed using 100 nearest neighbors, 50 PCs, and the ForceAtlas2 layout for visualization.

### Differential expression test

Differentially expressed genes within the sub-clusters found in Additional file 1: Fig. S9 and Additional file 1: Fig. S10 were calculated with the function *findMarkers* of the *scran R package* on log-transformed normalized counts. Genes with a false discovery rate below 0.05 were selected and then sorted by log2 fold change. In Figures S9e and S10e, genes with the top 20 highest/lowest values in the gene singular vectors are listed and colored blue if they correspond to the top 20 DE genes.

### Staining

A human fetal kidney (female) at week 15 of gestation was used for immunofluorescence using the same procedure as reported previously [30]. The following primary antibodies were used: rabbit anti-UPK1A (1:35, HPA049879, Atlas Antibodies), mouse anti-KRT7 (1:200, #MA5-11986, Thermo Fisher Scientific), rabbit anti-CDH1 (1:50, SC-7870, Santa Cruz), rabbit anti-CLDN1 (1:100, #717800, Thermo Fisher Scientific), goat anti-CAV2 (1:100, AF5788-SP, R&D Systems), mouse anti-AKAP12 (1:50, sc-376740, Santa Cruz), rabbit anti-CLDN11 (1:50, HPA013166, SIGMA Aldrich), mouse anti-POSTN (1:100, sc-398631, Santa Cruz), and goat anti-SULT1E1 (1:50, AF5545-SP, R&D Systems). The secondary antibodies were all purchased from Invitrogen and diluted to 1:500: Alexa Fluor 594 donkey anti-mouse (A21203), Alexa Fluor 594 donkey anti-rabbit (A21207), Alexa Fluor 647 donkey anti-mouse (A31571), Alexa Fluor 647 donkey anti-rabbit (A31573), and Alexa Fluor 647 donkey anti-goat (A21447). The sections were imaged on a Nikon Ti-Eclipse epifluorescence microscope equipped with an Andor iXON Ultra 888 EMCCD camera (Nikon, Tokyo, Japan).

## Supplementary Information

**Additional file 1:** Supplementary figures: Fig. S1 to Fig. S11.

**Additional file 2:** Contains further details on the mathematical derivation of phiclust.

**Additional file 3:** Contains a more detailed interpretation of the sub-clustering results for the human fetal kidney [46, 47].

**Additional file 4:** Review history.

**Additional file 5:** Table S1 Information on all scRNA-seq data sets and parameters for simulated data sets.

**Additional file 6:** Table S2 Tables with information on nuisance parameters.

**Additional file 7:** Table S3 Results of differential expression tests for BMNC data.

**Additional file 8:** Table S4 Results of differential expression tests for kidney data.

## Data Availability

All sequencing data sets were obtained from publicly available resources. The BMNC data can be downloaded with the R package SeuratData, named “bmcite.” The fetal kidney data is available in the GEO database under the accession number GSE114530. The PBMC data can be downloaded at https://cf.10xgenomics.com/samples/cell/pbmc3k/pbmc3k_filtered_gene_bc_matrices.tar.gz and the RNA-mix data is available at https://github.com/LuyiTian/sc_mixology, named “mRNAmix_qc”.
