# Supplementary material for: Phiclust: a clusterability measure for single-cell transcriptomics reveals phenotypic subpopulations
Source: Genome Biol. 2022 Jan 10;23:18. doi: 10.1186/s13059-021-02590-x (PMC8751334; doi:10.1186/s13059-021-02590-x)
Supplement: Supplementary file 1 — Additional file 1: Supplementary figures: Fig. S1 to Fig. S11. [file 13059_2021_2590_MOESM1_ESM.pdf]

Supplementary Figures

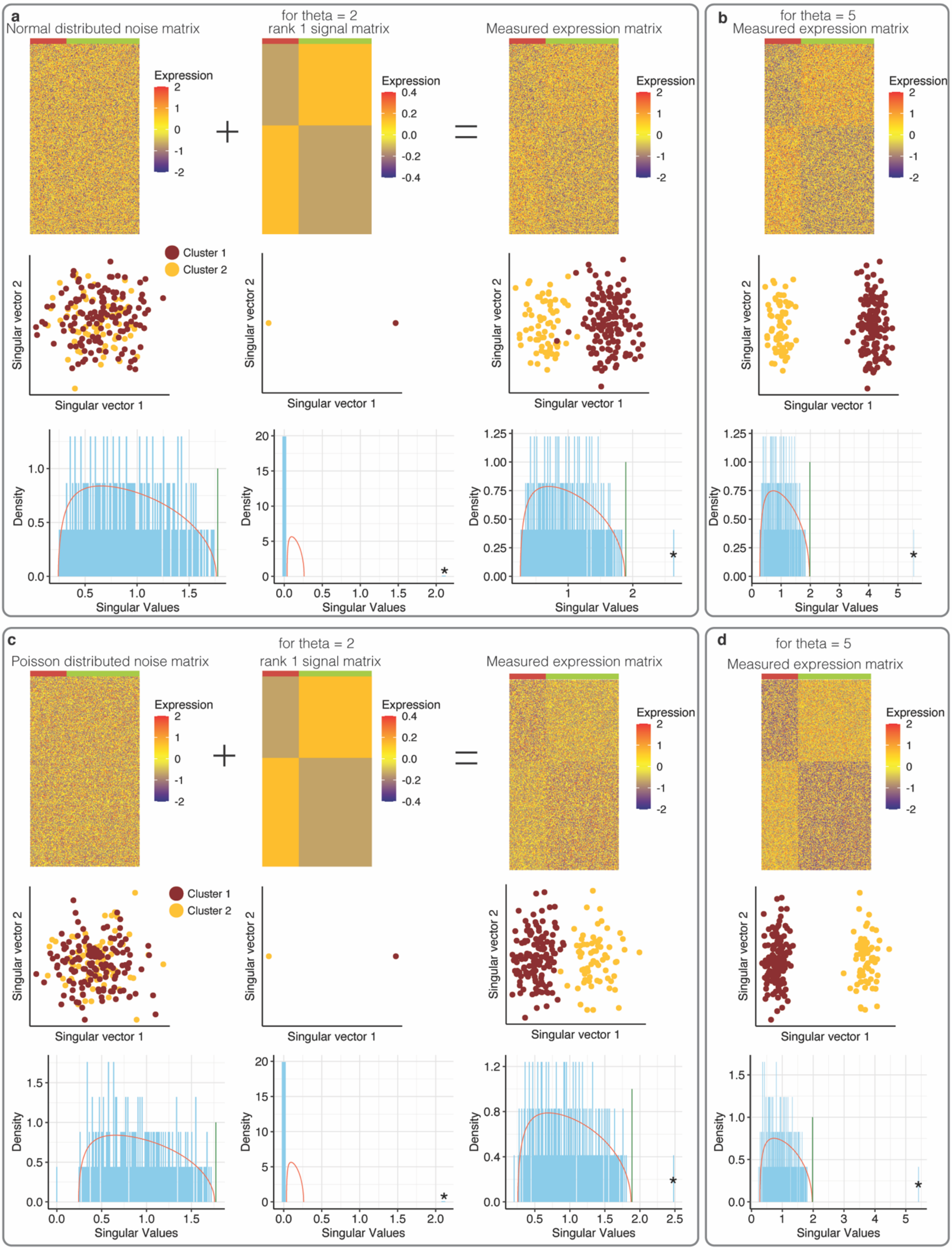

Fig. S1 | Basic principles of random matrix theory and perturbation theory

a Top row: heatmaps of a random matrix drawn from a normal distribution, a rank 1 signal matrix with a singular value θ of 2, and the resulting expression matrix. Middle row: Singular vectors of the corresponding matrices. Bottom row: histograms of the corresponding singular values. Red line: MP distribution, green line: TW threshold. **b** Heatmap, singular vectors and singular values of an expression matrix constructed as in a, except the singular value of the signal matrix was 5. **c,d** Matrices, singular vectors and singular values obtained as in a and b, but the random matrix was drawn from a Poisson distribution.

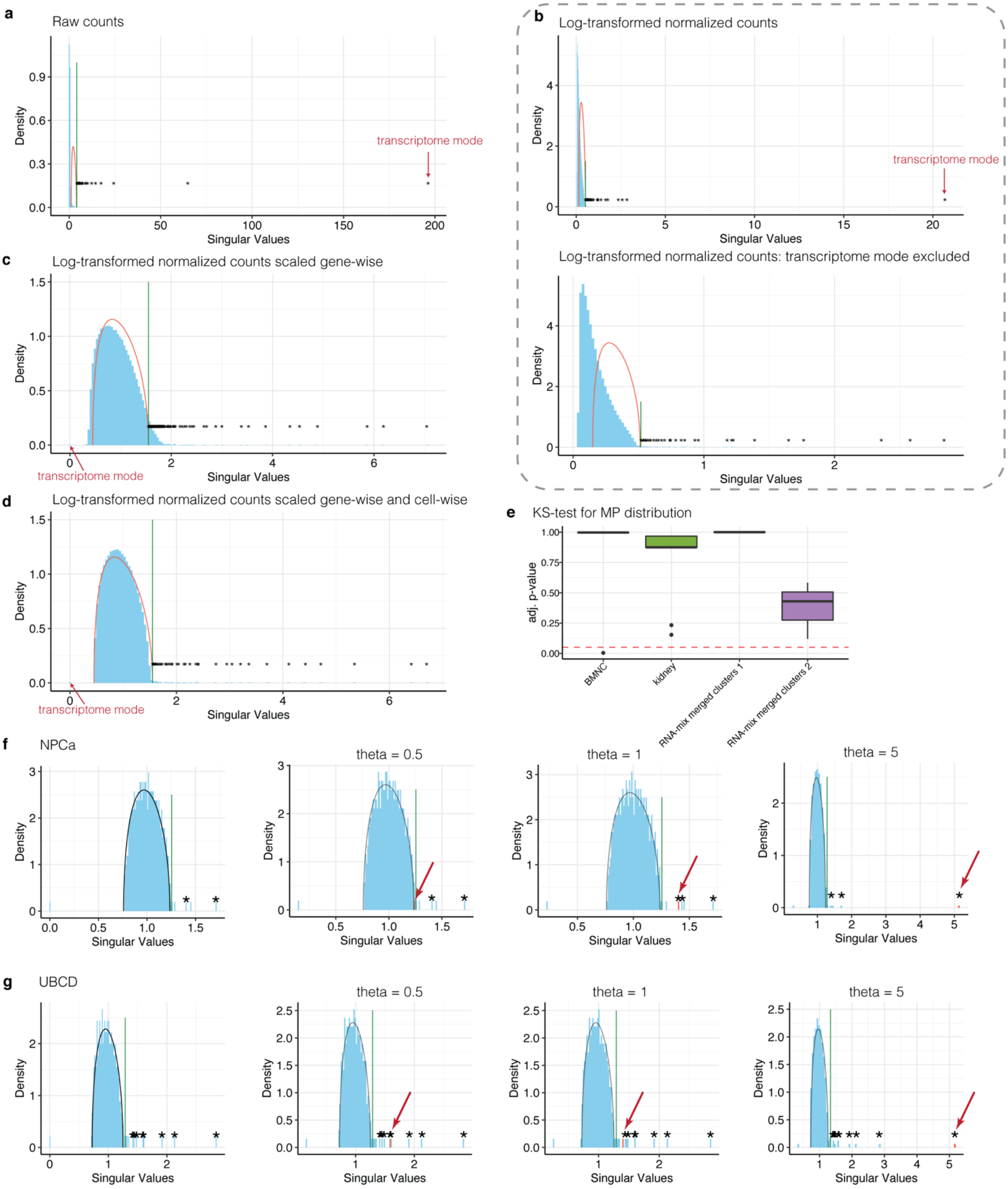
a
Raw counts
Density
Singular Values
transcriptome mode
b
Log-transformed normalized counts
Density
Singular Values
transcriptome mode
Log-transformed normalized counts: transcriptome mode excluded
Density
Singular Values
c
Log-transformed normalized counts scaled gene-wise
Density
Singular Values
transcriptome mode
d
Log-transformed normalized counts scaled gene-wise and cell-wise
Density
Singular Values
transcriptome mode
e
KS-test for MP distribution
adj. p-value
BMNC
kidney
RNA-mix merged clusters 1
RNA-mix merged clusters 2
f
NPCa
theta = 0.5
theta = 1
theta = 5
Density
Singular Values
g
UBCD
theta = 0.5
theta = 1
theta = 5
Density
Singular Values

Fig. S2 | Importance of preprocessing for MP fit and effect of perturbation on singular value distribution

Singular value (SV) distributions of the fetal kidney single-cell RNA-seq data set after different preprocessing steps. **a** Raw UMI counts. Arrow indicates transcriptome mode **b** Log-transformed, normalized UMI counts. Arrow indicates transcriptome mode. Right: Transcriptome mode was excluded. **c** Log-transformed, -normalized data as in b, that was additionally centered gene-wise. The transcriptome mode, visible as the highest singular value in a and b appears close to 0 (indicated by the arrow). **d** Log-transformed, normalized, and gene-wise standardized data, as in c, that was additionally standardized cell-wise. The SV distribution coincides with the bulk of the MP distribution. This is not a fit: The MP distribution is completely determined by the dimensions of the matrix and has no free parameters. **e** Kolmogorov-Smirnov (KS) test of a significant difference between the bulk of the singular value distributions and the MP distribution. The boxplot shows the adjusted p-values of the KS test for each cluster per data set. Red dashed line indicates an adjusted p-value of 0.05. **f** Histogram of singular values for the NPCa cluster of the fetal kidney data set. Left: original values. Rest: Singular values of the NPCa expression matrix plus a rank 1 perturbation with increasing magnitudes of the perturbation (singular value θ of the perturbation = 0.5, 1 or 5). The red arrow indicates the singular value that stems from the additional perturbation. **g** Histogram of singular values for the UBCD cluster of the fetal kidney data set. Left: original values. Rest: Singular values of UBCD expression matrix plus a rank 1 perturbation with increasing magnitudes of perturbation (singular value θ of the pertrubation = 0.5, 1 or 5). The red arrow indicates the singular value that stems from the additional perturbation.

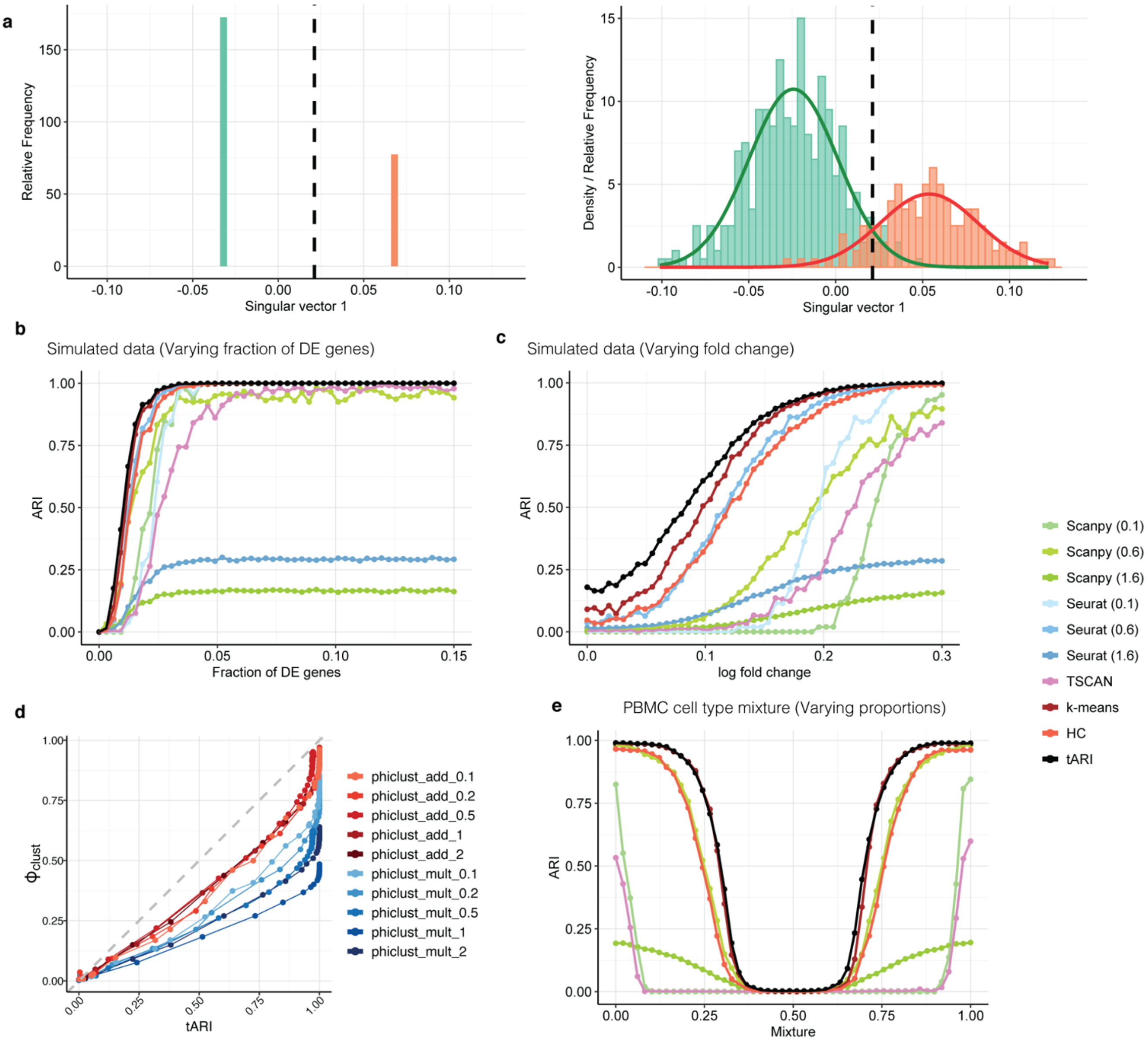

a
Relative Frequency
Singular vector 1
Density / Relative Frequency
Singular vector 1
b
Simulated data (Varying fraction of DE genes)
ARI
Fraction of DE genes
c
Simulated data (Varying fold change)
ARI
log fold change
Scanpy (0.1)
Scanpy (0.6)
Scanpy (1.6)
Seurat (0.1)
Seurat (0.6)
Seurat (1.6)
TSCAN
k-means
HC
tARI
d
Φclust
tARI
phiclust_add_0.1
phiclust_add_0.2
phiclust_add_0.5
phiclust_add_1
phiclust_add_2
phiclust_mult_0.1
phiclust_mult_0.2
phiclust_mult_0.5
phiclust_mult_1
phiclust_mult_2
e
PBMC cell type mixture (Varying proportions)
ARI
Mixture

Fig. S3 | An upper limit to the achievable ARI can be estimated using a Bayes classifier.

a Left: Histogram of the noise-free singular vector for a scenario with two clusters (or phenotypes) . Only the first singular vector is significant. The dashed line indicates a possible decision boundary. Right: Histogram of the first singular vector in the presence of noise. The color indicates to which simulated (ground truth) cluster the cells belong. Two normal distributions fitted separately to the singular vector entries belonging to the two clusters are shown as solid lines. The Bayesian error rate is estimated from the overlap of these two distributions and used to calculate the theoretical ARI (tARI). The dashed line indicates the optimal decision boundary. **b** ARI achieved by various clustering methods compared to the ground truth and tARI for simulated data with two clusters. The number of differentially expressed genes was varied. **c** ARI achieved by various clustering methods compared to the ground truth and tARI for simulated data with two clusters. The mean log fold change between clusters was varied. **d** tARI versus phiclust for simulated data sets with two clusters and different fractions of DE genes. Red curves: Values of phiclust for additive perturbation at different cell to gene ratios. Blue curves: Values of phiclust for multiplicative perturbation at different cell to gene ratios. Dashed grey line indicates diagonal. **e** ARI achieved by various clustering methods compared to the ground truth and tARI for PBMC cell type mixtures. Two synthetic clusters were created by weighted averages of cells from two clusters in the PBMC data set (see Fig. 1d). The mixture proportions were varied from 0 to 1. **b**,**c,e** The numbers in the legend indicate the resolution parameter used.

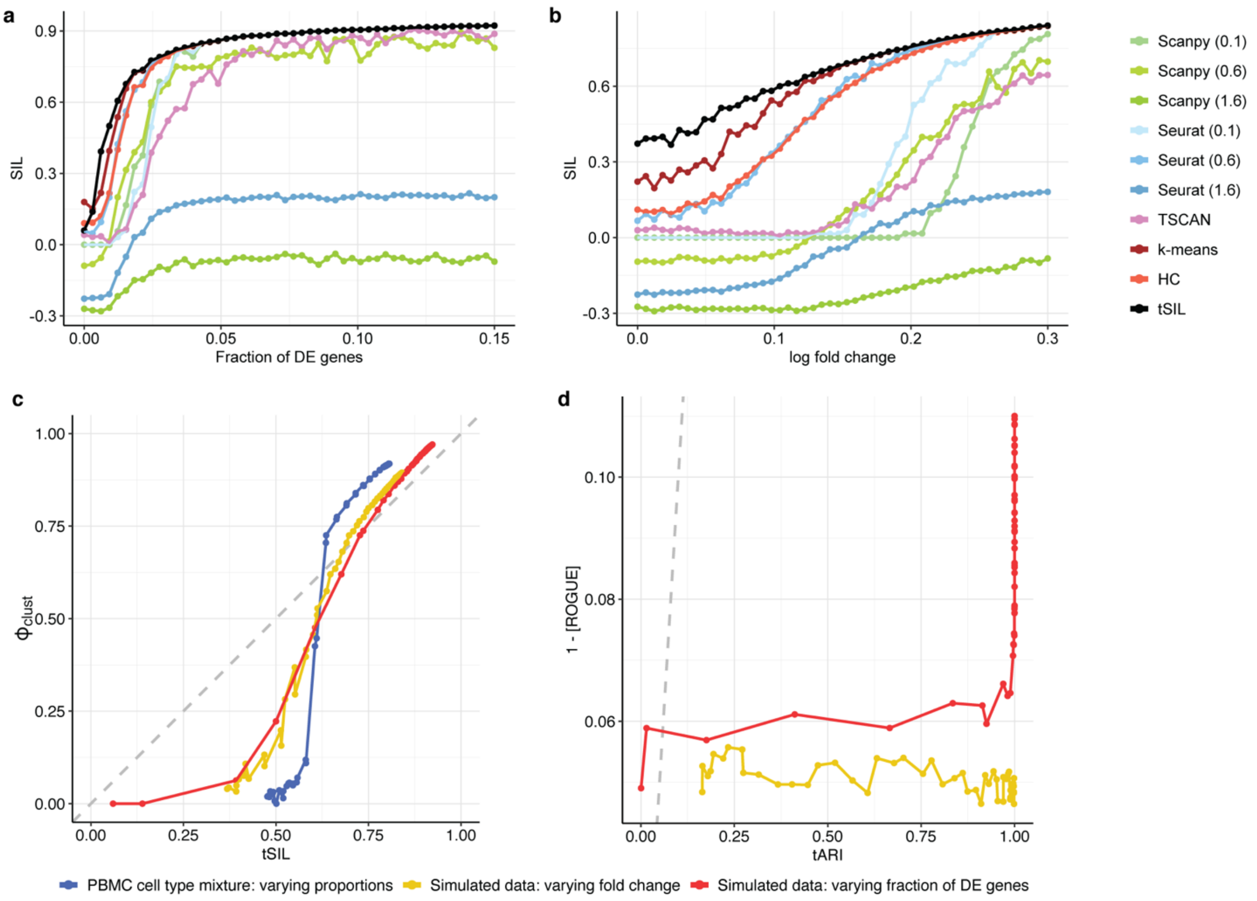

Fig. S4 | An approximate upper limit to the best possible silhouette coefficient and accordance of ROGUE with tARI.

a Silhouette coefficient (SIL) achieved by various clustering methods and theoretical SIL (tSIL) for simulated data with two clusters. The number of differentially expressed (DE) genes was varied. **b** SIL achieved by various clustering methods and tSIL for simulated data with two clusters. The mean log fold change between clusters was varied. **a**,**b** The numbers in the legend indicate the resolution parameter used. **c** tSIL versus phiclust. Red data points: Simulated data sets with two clusters. The number of DE genes was varied, the log fold change between clusters was fixed. Green data points: Simulated data sets with two clusters. The log fold change between clusters was varied, the number of DE genes was fixed. Blue data points: Two synthetic clusters were created by weighted averages of cells from two clusters in the PBMC data set (see Fig. 1d). Cluster weights were varied. The Grey dashed line indicates identity. **d** tARI versus 1 - [ROGUE] score. Red data points: Simulated data sets with two clusters. The number of DE genes was varied, the log fold change between clusters was fixed. Green data points: Simulated data sets with two clusters. The log fold change between clusters was varied, the number of DE genes was fixed. Blue data points: Two synthetic clusters were created by weighted averages of cells from two clusters in a PBMC data set (see Fig. 1d). Cluster weights were varied. The Grey dashed line indicates identity.

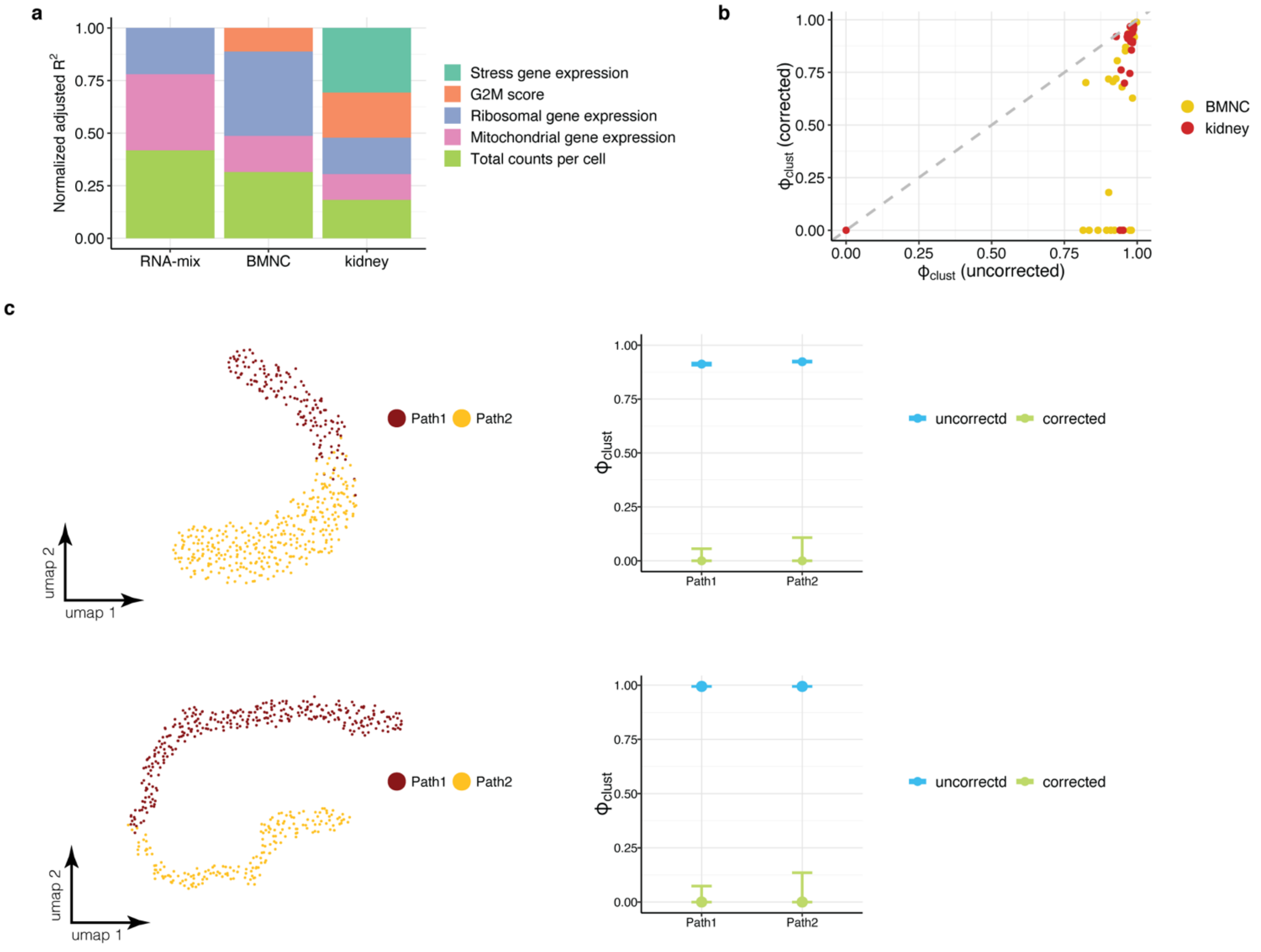

Fig. S5 I Correcting for nuisance parameters and unwanted variability.

a Summary of adjusted R^2 for several nuisance parameters in all experimental data sets. **b** Original (uncorrected) phiclust values vs phiclust corrected for the influence nuisance parameters in the BMNC and fetal kidney data sets. Dashed grey line indicates diagonal. **c** Two examples of differentiation paths with different numbers of differentially expressed genes. Left: UMAPs of simulated data sets with two differentiation paths. Right: Original (uncorrected) values of phiclust and phiclust values corrected by confounder regression using pseudotime as the only confounder.

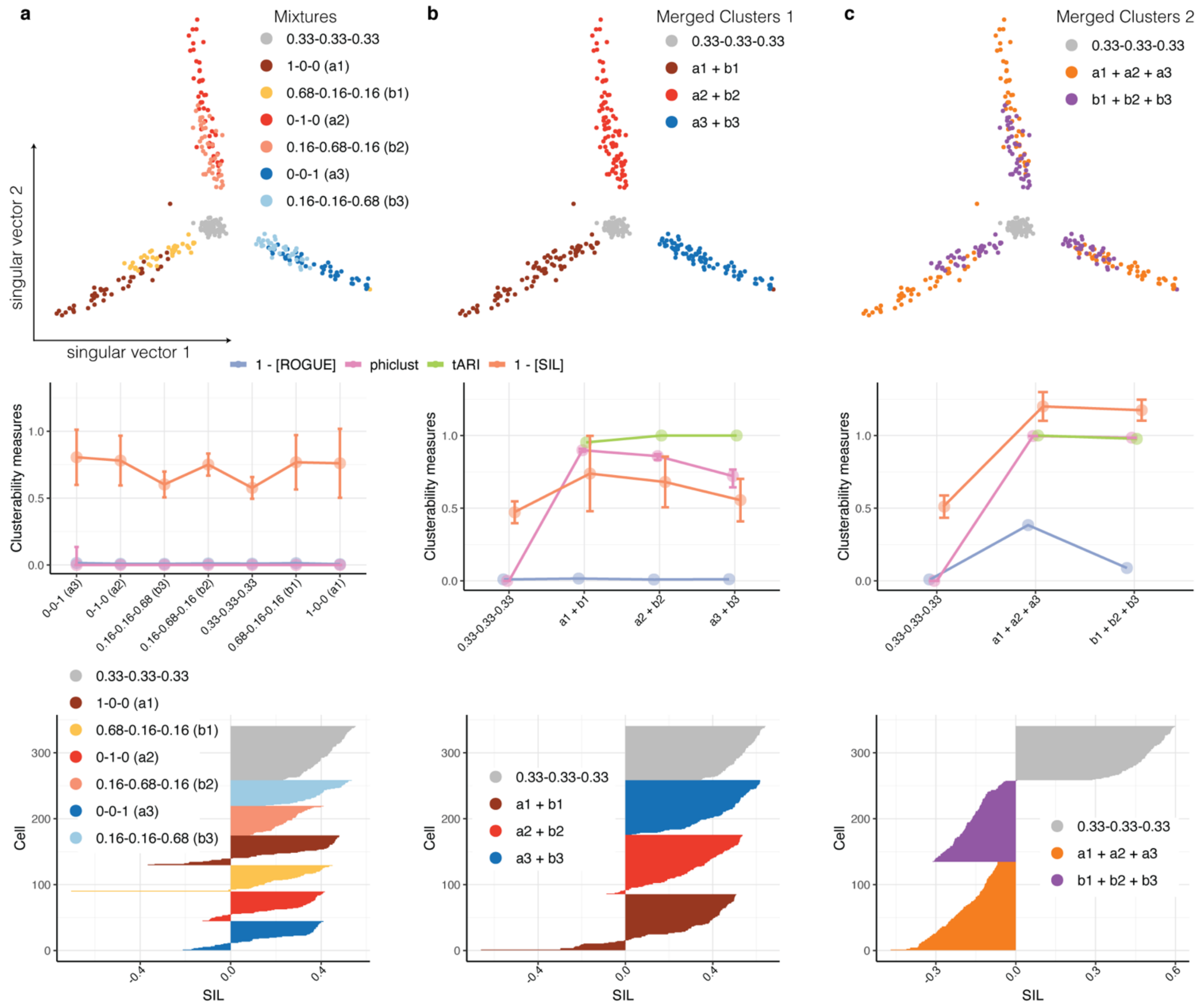

Fig. S6 | Phiclust outperforms other measures on experimental data.

Clusters of mixtures of RNA extracted from three different cell lines were merged in different ways to vary the amount of variability in each merged cluster. Top: first two singular vectors of RNA mixture data. Colors indicate different ratios of contributions from the three cell lines. Middle: The values of phiclust (rose), 1 – silhouette coefficient [SIL] (orange), tARI (green) and 1 - [ROGUE] (blue) for each corresponding cluster. For the calculation of the error bars, see Methods. Bottom: Bar plot of silhouette coefficients for each cell, sorted by cluster. **a** Original RNA mixture. **b** Merged clusters. Red: 0-1-0 merged with 0.16-0.68-0.16. Blue: 0-0-1 merged with 0.16-0.16-0.68. Green: 1-0-0 merged with 0.68-0.16-0.16. **c** Violet: merged cluster contains mixtures 0.68-0.16-0.16, 0.16-0.68-0.16 and 0.16-0.16-0.68. Orange: merged cluster contains mixtures 1-0-0, 0-1-0, and 0-0-1.

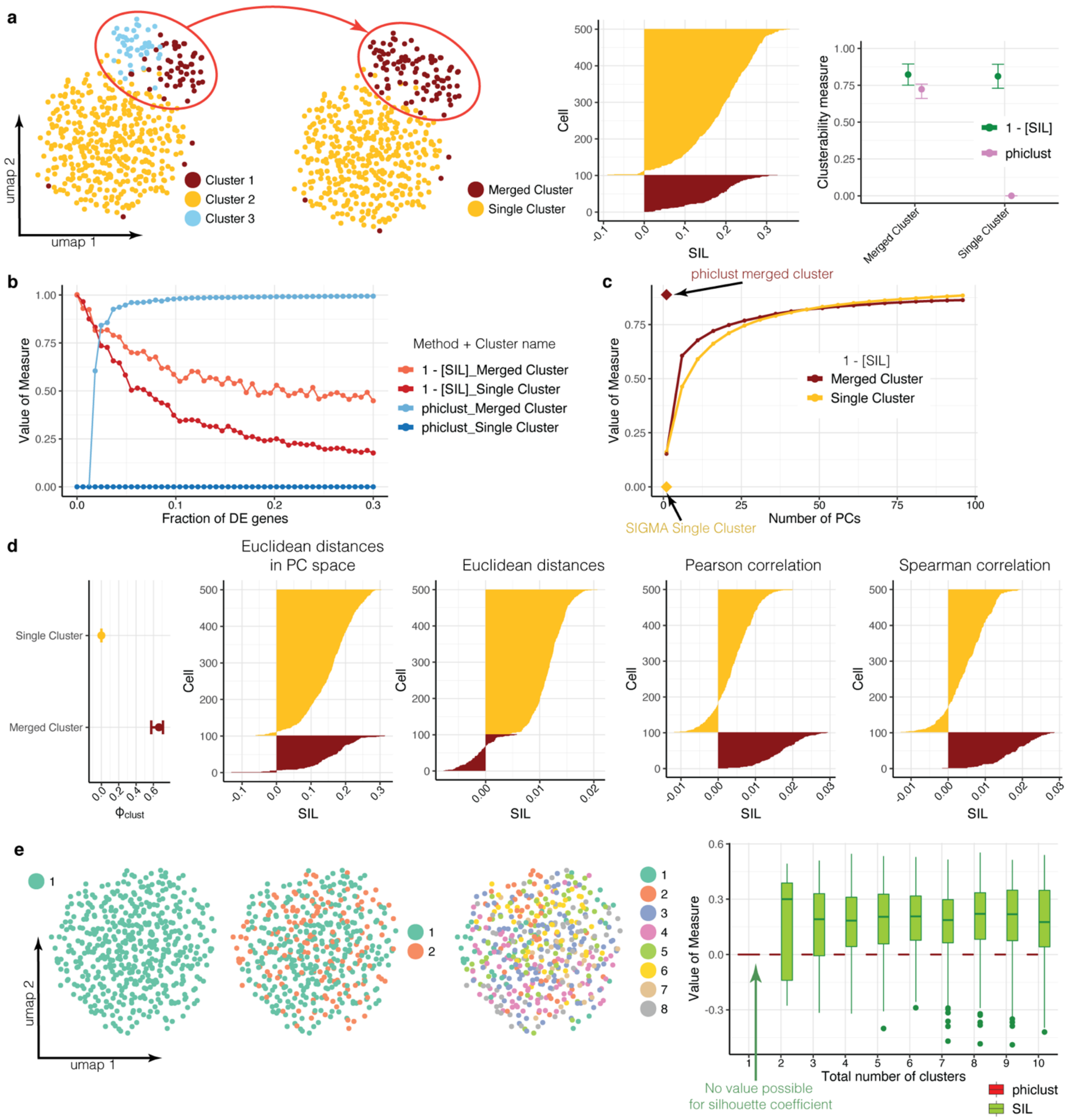

a
Cluster 1
Cluster 2
Cluster 3
umap 1
umap 2
Merged Cluster
Single Cluster
Cell
SIL
Clusterability measure
1 - [SIL]
phiclust
b
Value of Measure
Fraction of DE genes
Method + Cluster name
1 - [SIL]_Merged Cluster
1 - [SIL]_Single Cluster
phiclust_Merged Cluster
phiclust_Single Cluster
c
phiclust merged cluster
1 - [SIL]
Merged Cluster
Single Cluster
Number of PCs
SIGMA Single Cluster
d
Euclidean distances in PC space
Euclidean distances
Pearson correlation
Spearman correlation
Single Cluster
Merged Cluster
Φ_{clust}
e
Total number of clusters
No value possible for silhouette coefficient
phiclust
SIL

Fig. S7 | Phiclust outperforms the silhouette coefficient on simulated data.

a Left: UMAP of 3 simulated clusters. Two clusters were merged to one, resulting in two clusters in total. Middle: Bar plot of silhouette coefficients for each cell, sorted by cluster. Right: phiclust value and average silhouette coefficient for each cluster. **b** Simulation of clusters as shown in **a** with different fractions of differentially expressed genes. Phiclust (blue) and average silhouette coefficient (red) for merged cluster and single cluster. **c** Simulation of clusters as shown in **a**. The average silhouette coefficient per cluster was calculated for Euclidean distances using different numbers of principal components. Value of phiclust for each cluster is indicated by diamond-shaped data points. **d** Simulation of clusters as shown in **a**. Leftmost graph: Value of phiclust for each cluster. Other graphs: Bar plot of silhouette coefficients for each cell, sorted by cluster, calculated with the following distance metrics: Euclidean distances in principal component space, Euclidean distances in the original space, Pearson correlation and Spearman correlation. **e** Simulation of a cluster consistent with random noise. Clustering was performed with k-means to obtain 2 to 10 clusters. Left: UMAP of simulated data with 0, 2 and 8 clusters. Right: Boxplot of the values of phiclust and the silhouette coefficient for each k-means clustering.

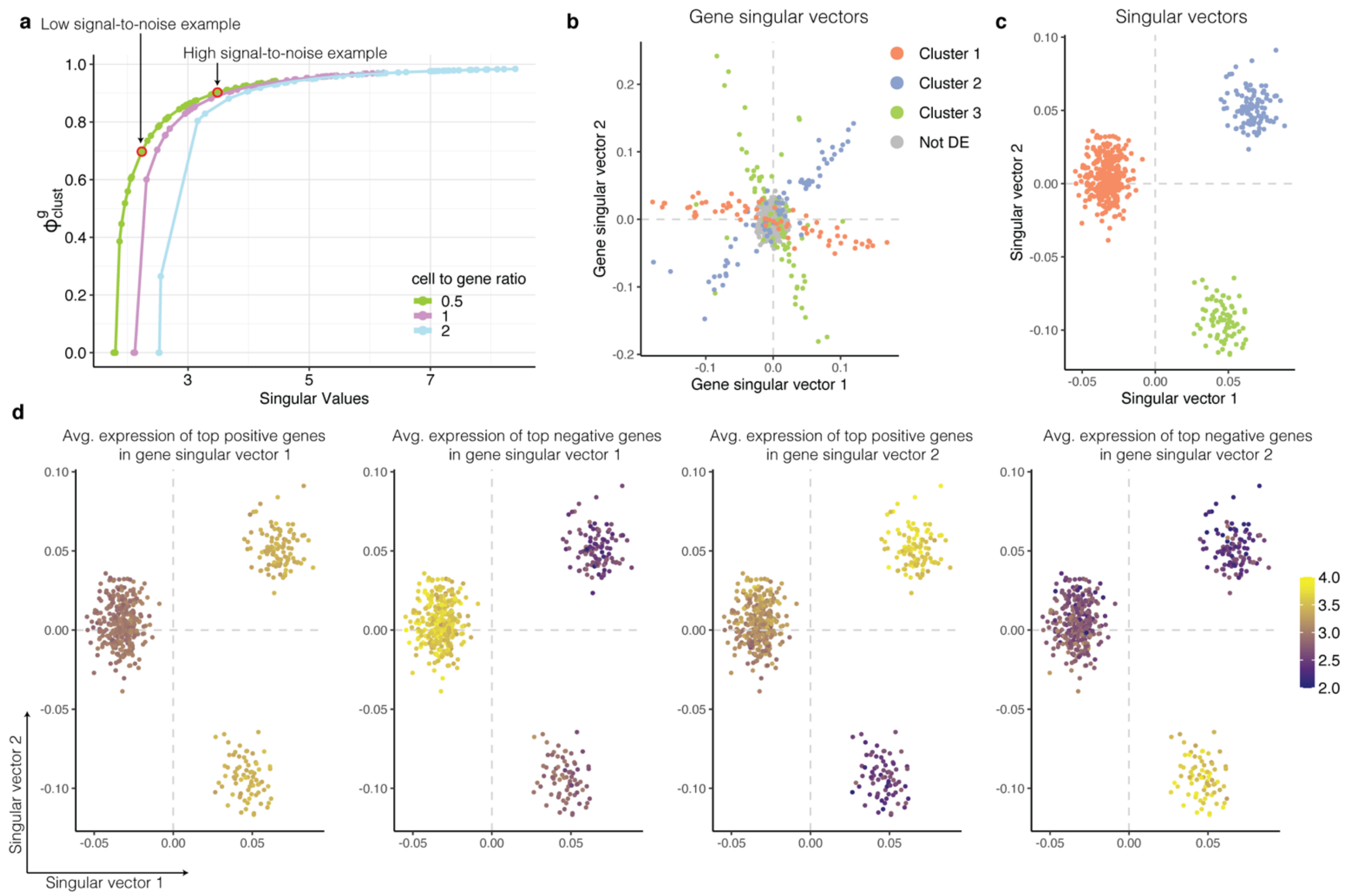

Fig. S8 | Variance-driving genes identified in gene singular vectors coincide with differentially expressed genes in a simulated data set.

Genes with high absolute values in the gene singular vector contribute the most to the variability. **a** Value of the largest singular value versus the squared cosine of the angle between the gene singular vector of the signal matrix and the gene singular vector of the measured expression matrix (g-phiclust) in simulated data. Arrows indicate examples shown in Figure 1b. **b** First two gene-singular vectors. Differentially expressed genes of each cluster are indicated by color. **c** First two (cell-)singular vectors for the simulated data set shown in panel b. Dashed grey lines indicate the 0 value on each of the axes. Cell clusters are indicated by color. **d** First two singular vectors as in c. Dashed grey lines indicate the 0 value on each of the axes. The average log-transformed expression of the top 1% genes driving the variance is indicated by color. The 4 panels show, respectively, from left to right: genes corresponding to the highest values in gene singular vector 1, genes corresponding to the lowest values in gene singular vector 1, genes corresponding to the highest values in gene singular vector 2, and genes corresponding to the lowest values in gene singular vector 2.

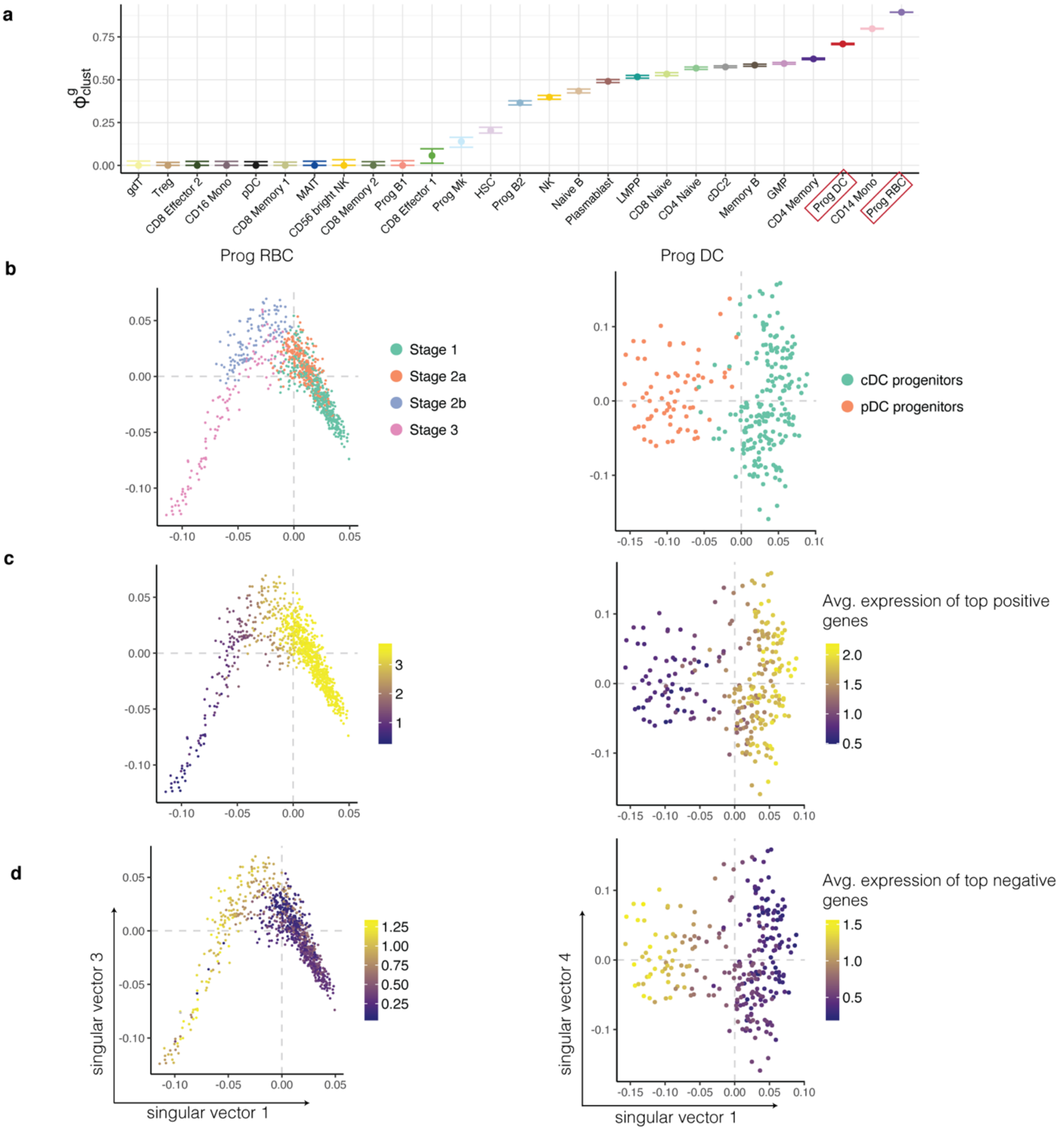

e

top positive genes Stage1 / Stage2a	top negative genes Stage2b / Stage3
RPS2	HBA2
RPL37A	HBM
RPLP1	SNCA
PTMA	BPGM
RPS3	HBD
RPL27A	HBA1
RPLP0	SLC4A1
RPL3	ALAS2
EEF1A1	TMCC2
RPS16	GYPB
RPL14	IFIT1B
RPL8	PDZK1IP1
RPL13A	SLC25A37
RPS18	TSPO2
RPS6	HEMGN
RPS9	KRT1
RPS23	HBB
RPS5	LGALS3
RPL36	BNIP3L
RPS17	NCOA4

● Genes in agreement with DE test

top positive genes cDC progenitors	top negative genes pDC progenitors
LYZ	IGLL1
S100A6	IRF8
S100A4	JCHAIN
LST1	TCF4
COTL1	ADA
CSTA	MZB1
TSPO	CCDC50
FTL	UGCG
MNDA	FAM129C
TMSB10	SCT
S100A10	NUCB2
S100A11	PLAC8
S100A9	ITM2C
CEBPD	IRF7
SPI1	STMN1
ANXA1	SPIB
RNASE2	SOX4
SH3BGRL3	APP
RPL8	C12orf75
ANXA2	TSPAN13

Fig. S9 | Congruence between variance-driving genes and differentially expressed genes between sub-clusters in a BMNC data set.

a g-phiclust for each cluster in the BMNC data set. **b** Singular vectors of the two clusters from the BMNC data set with the highest phiclust. The color indicates sub-clustering. Dashed grey lines indicate the 0 value on each of the axes. **c** Singular vectors of clusters shown in panel a with color indicating the average log-transformed gene expression of genes with the 1% highest values in the first gene singular vector. **d** Singular vectors of clusters shown in panel a with color indicating the average log-transformed gene expression of genes with the 1% lowest values in the first gene singular vector. **e** Genes driving the variance in the two clusters shown in b. These genes have the 20 highest/lowest values in the first gene singular vector respectively. In blue: top 20 upregulated genes based on differential expression (DE) test between the sub-clusters using *findMarkers* (from *scran* R package).

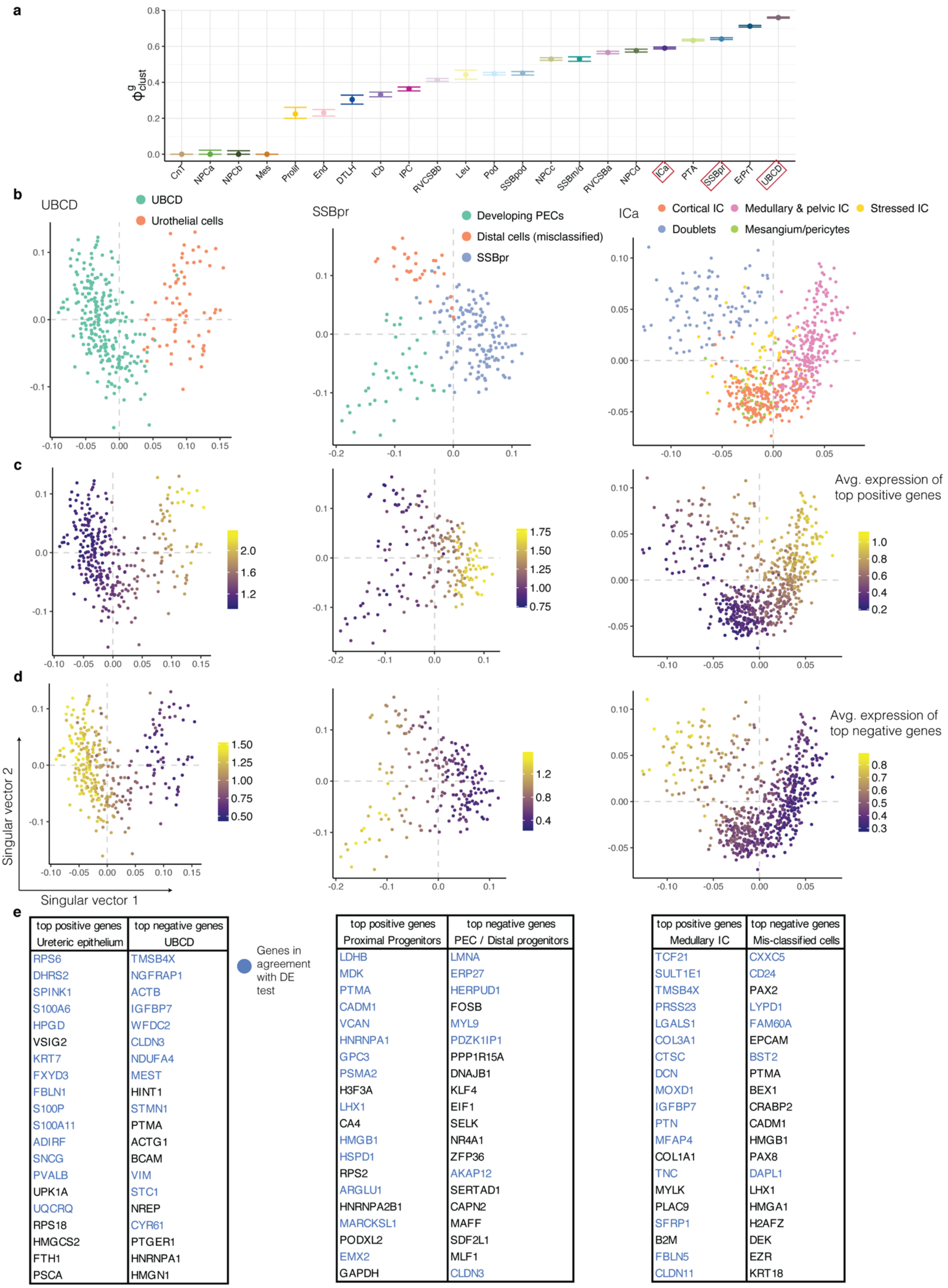

e

top positive genes Ureteric epithelium	top negative genes UBCD
RPS6	TMSB4X
DHRS2	NGFRAP1
SPINK1	ACTB
S100A6	IGFBP7
HPGD	WFDC2
VSIG2	CLDN3
KRT7	NDUFA4
FXYD3	MEST
FBLN1	HINT1
S100P	STMN1
S100A11	PTMA
ADIRF	ACTG1
SNCG	BCAM
PVALB	VIM
UPK1A	STC1
UQCRQ	NREP
RPS18	CYR61
HMGCS2	PTGER1
FTH1	HNRNPA1
PSCA	HMGN1

Genes in agreement with DE test

top positive genes Proximal Progenitors	top negative genes PEC / Distal progenitors
LDHB	LMNA
MDK	ERP27
PTMA	HERPUD1
CADM1	FOSB
VCAN	MYL9
HNRNPA1	PDZK1IP1
GPC3	PPP1R15A
PSMA2	DNAJB1
H3F3A	KLF4
LHX1	EIF1
CA4	SELK
HMGB1	NR4A1
HSPD1	ZFP36
RPS2	AKAP12
ARGLU1	SERTAD1
HNRNPA2B1	CAPN2
MARCKSL1	MAFF
PODXL2	SDF2L1
EMX2	MLF1
GAPDH	CLDN3

top positive genes Medullary IC	top negative genes Mis-classified cells
TCF21	CXXC5
SULT1E1	CD24
TMSB4X	PAX2
PRSS23	LYPD1
LGALS1	FAM60A
COL3A1	EPCAM
CTSC	BST2
DCN	PTMA
MOXD1	BEX1
IGFBP7	CRABP2
PTN	CADM1
MFAP4	HMGB1
COL1A1	PAX8
TNC	DAPL1
MYLK	LHX1
PLAC9	HMGA1
SFRP1	H2AFZ
B2M	DEK
FBLN5	EZR
CLDN11	KRT18

Fig. S10 | Congruence between variance-driving genes and differentially expressed genes between sub-clusters in a fetal kidney data set.

a g-phiclust for each cluster in the fetal kidney data set. **b** First two singular vectors of three clusters from the fetal kidney data set with high phiclust. The color indicates sub-clustering. Dashed grey lines indicate the 0 value on each of the axes. **c** First two singular vectors of clusters shown in panel a with color indicating the average log-transformed gene expression of genes with the 1% highest values in the first gene singular vector. **d** First two singular vectors of clusters shown in panel a with color indicating the average log-transformed gene expression of genes with the 1% lowest values in the first gene singular vector. **e** Genes driving the variance in the three clusters shown in b. These genes have the 20 highest/lowest values in the first gene singular vector respectively. In blue: top 20 upregulated genes based on differential expression (DE) test between the sub-clusters using *findMarkers* (from *scran* R package).

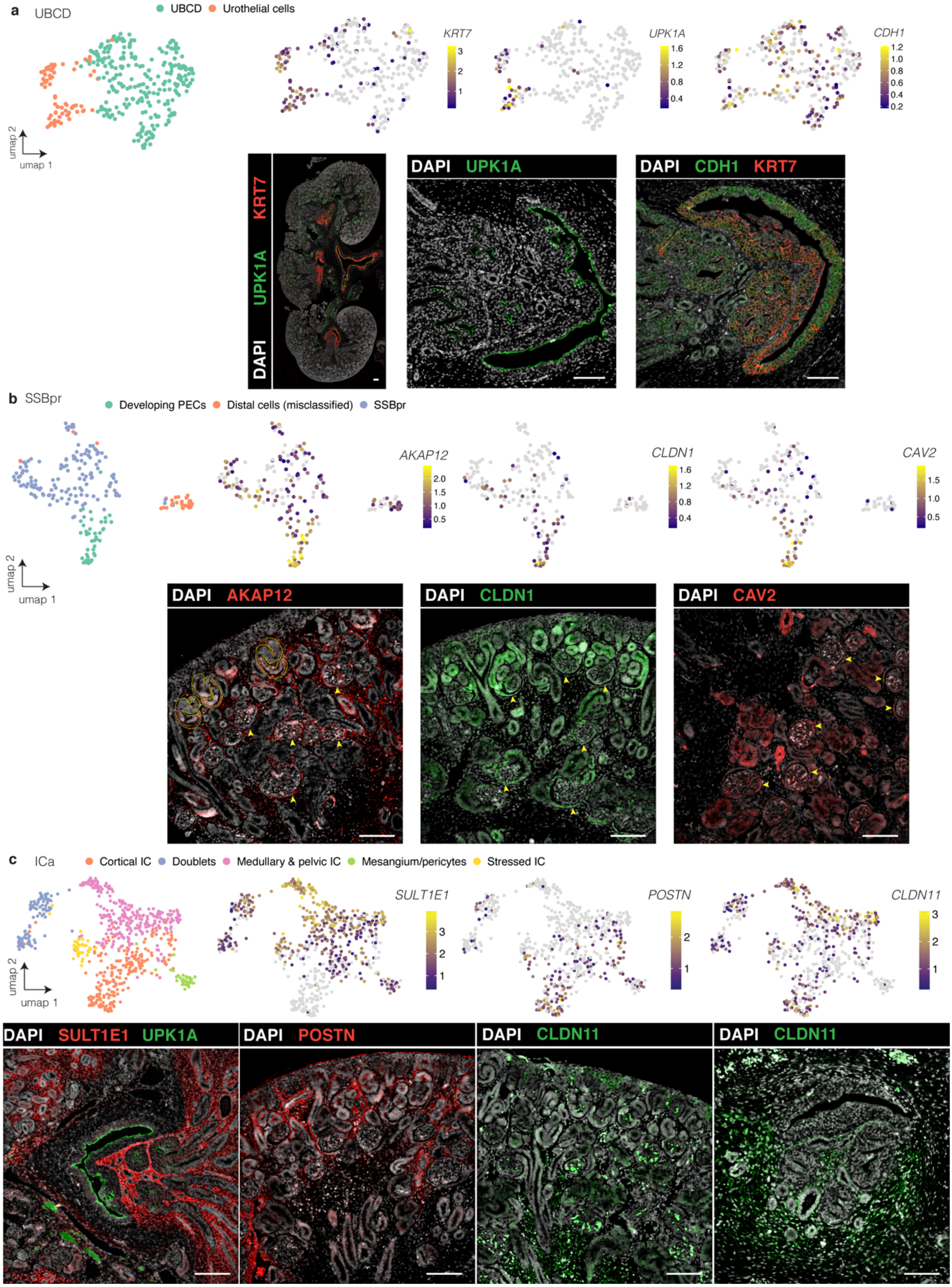
a
UBCD
UBCD
Urothelial cells
umap 2
umap 1
KRT7
UPK1A
CDH1
DAPI UPK1A KRT7
DAPI UPK1A
DAPI CDH1 KRT7
b
SSBpr
Developing PECs
Distal cells (misclassified)
SSBpr
AKAP12
CLDN1
CAV2
DAPI AKAP12
DAPI CLDN1
DAPI CAV2
c
ICa
Cortical IC
Doublets
Medullary & pelvic IC
Mesangium/pericytes
Stressed IC
SULT1E1
POSTN
CLDN11
DAPI SULT1E1 UPK1A
DAPI POSTN
DAPI CLDN11
DAPI CLDN11

Fig. S11 I Immunostaining validates newly identified subclusters in fetal kidney data set.

a-c Upper panels show UMAPs of the selected clusters in the fetal kidney data set. Log-normalized expression of selected genes is indicated by color. Lower panels show immunostainings of week 15 fetal kidney sections. **a** UBCD cluster. UPK1A, CDH1, and KRT7 expression is shown in a complete section (leftmost image) and in the urothelial epithelium. **b** SSBpr cluster. Expression of AKAP12, CLDN1 and CAV2 is shown. The dashed lines indicate S-shaped bodies, arrowheads indicate PECs in developing glomeruli **c** ICa cluster. Expression of SULT1E1 and UPK1A is visible around the ureter expression of POSTN is visible in cortical areas, CLDN11 is visible in the cortical area (CLDN11, left image) and around the ureter (CLDN11, right image). Scale bars: 100 μm.
