# Supplementary material for: Phiclust: a clusterability measure for single-cell transcriptomics reveals phenotypic subpopulations
Source: Genome Biol. 2022 Jan 10;23:18. doi: 10.1186/s13059-021-02590-x (PMC8751334; doi:10.1186/s13059-021-02590-x)
Supplement: Supplementary file 3 — Additional file 3: Contains a more detailed interpretation of the sub-clustering results for the human fetal kidney [46, 47]. [file 13059_2021_2590_MOESM3_ESM.pdf]

Additional file 3

Application of phiclust to our previously published single-cell RNA-sequencing study of the human fetal kidney [30] revealed two distinct groups of clusters (Fig. 3a). Connecting tubule (CnT), nephron progenitor cells-a (NPCa), nephron progenitor cells-b (NPCb), and mesangial cells (Mes) all obtained a phiclust of 0, which signified that these clusters consist of pure populations with homogeneous gene-expression profiles. The rest of the clusters obtained higher values of phiclust, indicating that they contained subpopulations that were previously overlooked. For further analysis, we explored all clusters with highest phiclust and chose to further investigate clusters in which new cell populations were identified: the ureteric bud/collecting duct (UBCD), the S-Shaped Body proximal precursor cells (SSBpr), and the Interstitial cells a (ICa), with phiclust of 0.97, 0.95, and 0.93, respectively.

UBCD

The analysis of this cluster yielded two clearly separate subpopulations (Fig. 3b, Additional file 1: Fig. S10b). The bigger subpopulation contained developing collecting duct cells and their precursors (ureteric bud), indicated by the expression of genes such as *WFDC2*, *AQP2*, *CLDN3*, *MMP7,* and *CALB1.* In contrast, the smaller sub-cluster showed little or no expression of the aforementioned genes and was characterized by *UPK1A* and *UPK1B,* well-known markers of the urothelial epithelium, which constitutes the inner lining of the ureter. The presence of such cells in our data is plausible given that the whole fetal kidney was used in our sequencing experiment. Both DE analysis (Table S4) and inspection of the top variance-driving genes (Additional file 1: Fig. S10e) revealed *SPINK1*, *UPK2*, *S100A6*, *KRT7,* and *KRT19* as additional markers. Staining of week 15 fetal kidney sections with UPK1A and KRT7 antibodies confirmed our interpretation (Fig. 3c, Additional file 1: Fig. S11a). UPK1A was restricted to the superficial urothelial cells in major and minor calyces as well as the developing ureter. KRT7 was expressed more broadly, across the superficial, intermediate, and basal urothelium. Both KRT7 and UPK1A were completely absent from the whole collecting system and the branching ureteric bud, marked by CDH1 (Additional file 1: Fig. S11a).

SSBpr

Sub-clustering the SSBpr population showed the presence of 3 subpopulations (Fig. 3b, Additional file 1: Fig. S10b). One subpopulation contained markers of proximal cell precursors (*GPC3*, *LHX1*, *CADM2*) together with low expression of *AMN* and *APOE* (see Table S4), which is consistent with the original annotation of the cluster.

A second subpopulation, contiguous to the previous one, showed the expression of *CLDN1*, which is expressed in the proximal epithelium, together with *CITED2*, expressed in developing podocytes. This suggested parietal epithelial cells (PECs) as the most likely cell type, as these cells were reported to share several markers with both proximal epithelium and podocytes [46]. To confirm this interpretation, we performed Immunostaining of CLDN1, as well as CAV2 and AKAP12 which were found by DE analysis (Fig. 3d, Additional file 1: Fig. S11b). Interestingly, CLDN1 was found in all segments of the S-shaped body except in the precursors of the PECs, which are the thin layer of cells at the lateral side of the proximal segment of the SSB. CLDN1 appeared in the parietal epithelium only at the capillary loop stage and continued to be expressed in all PECs in more mature glomeruli. CAV2 was present in the parietal epithelium in developing glomeruli, but also in the endothelial cells of both the glomerular capillaries and the surrounding vasculature. Intriguingly, CAV2 overlapped with CLDN1 only in a subpopulation of PECs in individual glomeruli, which might indicate previously unobserved heterogeneity within these cells in the developing kidney. Only AKAP12 marked the precursors of the PECs in S-shaped bodies and continued to

be abundantly expressed. However, AKAP12, was not specific to PECs, as it was also expressed in interstitial cells in the cortex.
Finally, a third, small and distinct subpopulation in the SSBpr cluster expressed distal tubule markers (*SPP1*, *ODC1*, *IRX3,* and *S100A10*), suggesting that these cells were misclassified during the original clustering. This shows that phiclust can pinpoint clustering errors, making it a useful tool for clustering quality control.

ICa

This cluster consisted of 5 subpopulations (Fig. 3b, Additional file 1: Fig. S10b). All subpopulations expressed markers of the renal interstitium. One also expressed genes found uniquely expressed in other cell types (*EPCAM*, *CD24*, *BST2*, *NNAT*, *DAPL1*) and thus likely contains doublets. Another small subset was characterized by markers of mesangial cells (*MGP*, *ACTA2*, *PDGFRB*), suggesting that it contains mesangial cells erroneously grouped with the ICa or renal pericytes, which share a similar gene expression profile [47]. Another subpopulation showed high expression of non-specific genes related to components and regulators of microtubules together with metabolic, mitochondrial and stress-related genes (*H2AFZ*, *TUBA1B*, *TYMS*, *STMN1*, *DUT*, *MT-CO3*, *MT-ND5*).
The two remaining subpopulations were clearly interstitial but their gene expression profiles could not be linked to known interstitial populations, likely due to the dearth of knowledge about the renal stroma. We hypothesized that these two subpopulations were localized in different regions of the kidney. To test this idea, we stained fetal kidney sections with POSTN, CLDN11, and SULT1E1 (Fig. 3e, Additional file 1: Fig. S11c), which were identified by DE analysis and inspection of the top variance-driving genes. SULT1E1 was highly expressed in the pelvic area in the immediate vicinity of the developing ureter, as well as the inner and outer medulla, preferentially surrounding tubules. This marker might thus indicate the medullary interstitium as well as pelvic smooth muscle cells. Staining with CLDN11 showed a higher signal in the medulla and papilla, similar to SULT1E1, but with a wider spatial distribution. In contrast to SULT1E1, CLDN11 was also expressed in groups of cortical interstitial cells, situated directly underneath the renal capsule, in the nephrogenic zone. CLDN11 might thus also be expressed by the interstitial progenitor cells or their immediate progeny. Lastly, POSTN was mainly found in the renal cortex surrounding tubules and glomerular microvasculature. POSTN was also expressed in cortical blood vessels with larger diameters together with their arborizations. POSTN is a secreted extracellular matrix protein known to be expressed in cardiac smooth muscle cells, as well as connective tissues. Here, POSTN might mark smooth muscle cells of the cortical vasculature.

In conclusion, a reanalysis of our previously published data showed the ability of phiclust to reveal overlooked subpopulations. Interestingly, phiclust identified sub-clusters with only a few cells (41 developing PECs, 68 urothelial cells, 29 distal cells), highlighting its sensitivity to relevant substructure hidden within a bigger cluster. Finally, phiclust was also useful to pinpoint clustering errors and the presence of doublets, which makes it useful for quality control prior to DE analysis.
