# Supplementary material for: Phiclust: a clusterability measure for single-cell transcriptomics reveals phenotypic subpopulations
Source: Genome Biol. 2022 Jan 10;23:18. doi: 10.1186/s13059-021-02590-x (PMC8751334; doi:10.1186/s13059-021-02590-x)
Supplement: Supplementary file 4 — Additional file 4: Review history. [file 13059_2021_2590_MOESM4_ESM.docx]

**1^st^ round**

**Reviewer 1**

The pre-print A clusterability measure for single-cell transcriptomics reveals phenotypic subpopulations proposes a new variable, SIGMA, for measuring the suitability of a given cell phenotype clustering from scRNA-seq data. To achieve this, the authors propose reinterpreting the signal-to-noise ratio of given clusters and sets of genes using random matrix theory. Under this formulation the background in e.g. the UMAP clusters is modelling as an MP distribution of the singular values of a random matrix, and it is possible to determine which genes are relevant for the true cell phenotype signal and what fraction of the observed clusters are explained by those signal genes by assessing the singular values above a given threshold on the MP background distribution. This analysis is statistically rigorous and uses well-researched results from random matrix theory. The major assumption is that noise enters the signal in an additive way, which is reasonable given the data.

This question of the suitability of scRNA-seq clusters is a very important one, and has not been tackled formally in many ways, making SIGMA a valuable technique for scRNA-seq analysis. As such, I believe this pre-print is a welcome addition to the field and addresses an important question in how we should be thinking about the quality of the cell types we determine using conventional machine learning methods in scRNA-seq analysis. My (mostly minor) comments are as follows:

1. L60: low-rank perturbation theory is introduced and referenced but I think it would be helpful to further explain its relevance to random matrix theory and scRNA-seq, particularly for readers not familiar with the method. I would do this by giving an example of the perturbations calculated from an example scRNA-seq data-set, and how this relates to e.g. the singular values obtained from the underlying MP distribution and TW bound.
2. L97: It is not clear how the Bayesian error rate corresponds to the lowest possible error in this application. Can you further explain and provide an example?
3. L122: The need to regress out nuisance parameters is a vital step in the SIGMA calculation, and as such I feel there should be a more extensive discussion of this step in the main text. I propose you move the Supplementary Figure 4, where the effects of the regression are shown, to the main body and describe it there. I would also add a table where the differences in the SIGMA value before and after correcting for the confounding nuisance parameters are stated for each data-set. It would also be interesting to comment on the relevant sizes of different nuisance parameters to the data (see comment 5).
4. L188-191: The choice of random matrix models assumes the noise is additive, which is justified given the results in this study with SIGMA. It would be helpful to quantify how good this assumption is by including a table of the closeness of singular values to the MP distribution, and indicate how much this changes between different data-sets. This way, it will be possible to make a rigorous statement about how much the MP behaviour varies between the different data-sets, and to confirm that the additive assumption of noise is valid for all data-sets in the study. Given the closeness of the behaviour to the MP distribution, it might also be possible to derive a modelling uncertainty on SIGMA. I think this would be useful as it can be directly translated into essentially a confidence interval on the results of the SIGMA clustering quality, where the size of the interval depends on the closeness of the MP modelling assumption to the truth.
5. L194: Does the hierarchy of the size of different confounding factors/nuisance parameters (RNA recovery vs. cell cycle phase dependence) vary between different data-sets, or is one effect dominating across most data-sets? If so, it would be interesting to quantify this effect and provide a possible explanation for why it is observed. A table of the various contributions of different nuisance parameters would be a welcome addition.

**Reviewer 2**

Upon reading the abstract, I found this topic to be interesting and useful for the single cell research community. However, upon reading the main text, I realized this manuscript has two major problems. First, a lot of the description of the method is too technical. I am a bioinformatician with sufficient exposure to statistics, and I still find it hard to understand this paper with too many technical jargons. This will be very hard for most bioinformaticians and almost all biologists who wish to use this tool. Secondly, the validation/application analyses are insufficient to really show this method's advantage. The validation/application practices mostly validated the clustering analyses done by the authors, but rather than SIGMA itself.
Major comments:
(1) Several places in the introduction need to be clarified to make sure readers understand the key logic flow of the story. (a) "Notwithstanding its usefulness, it cannot be excluded that a partition of random noise obtains a high silhouette coefficient, indicating high clustering quality." Doesn't SIGMA suffer from the same problem? (b) "We consider clusterability to be the theoretically achievable agreement with the unknown ground truth clustering, for a given signal-to-noise ratio." It will be important to introduce what is signal and what is noise here. (c) "Notions of clusterability have been proposed previously [6,9], but those are not directly compatible with scRNA-seq data". Need to state what exactly are the notions that have been proposed and why they are not compatible. Simply citing papers is not enough here.
(2) Same as above. "This is made possible by using universality properties of random matrix theory." Need some intuitive introduction on the random matrix theory and how it is used here. "We expand on these results by applying low-rank perturbation theory [13], which allows us to identify the signature of deterministic signals in the context of random noise." Same for "low-rank perturbation theory". And what do you mean by "deterministic signals"? "SIGMA is derived from first principles and does not have free parameters". What are "first principles" and "free parameters"?
(3) In Fig. S1, what is "transcriptome mode"? "If the data is preprocessed appropriately (Fig. S1)," After reading Fig. S1, I still don't quite get what constitutes "appropriate preprocessing". "The bulk of this distribution is described by the Marchenko-Pastur (MP) distribution, which corresponds to the random component of the measurement" Why? "we can calculate the angles between the singular vectors of the measured gene expression matrix and those of the (unobserved) signal matrix" How is this possible without knowing the unobserved signal matrix? Some intuition and laymen language need to be put into the main text, rather than stacked technical jargons.
(4) The same problem here: "Using a simple case of two clusters of cells with varying signal-to-noise ratios, we estimated the Bayesian error rate [18] (i.e. the lowest possible error) for this binary classification problem in simulated data (Fig. S2a). Based on this error rate, we calculated a theoretically achievable ARI (tARI, see also Supplementary Note 1). We showed empirically that commonly used clustering methods do not exceed this limit (Fig. S2b,c)." The authors need to point out intuitively what has been done, instead of simply pointing to a figure or supplemental material. For example, what is signal and what is noise in this sentence: "varying signal-to-noise ratios". After reading this part, I am totally unsure what was done and whether it is correct or not.
(5) "We noticed that the amount of input RNA used for each mixture was a confounding factor that influenced the value of SIGMA (Fig. S4b,c)." This seems to be a critical problem affecting SIGMA. Why would the amount of input RNA affect clustering results? Usually, the expression counts of each cell were normalized to something like TPM, and theorectically, the amount of input RNA will already be irrelevant after this step, and should not affect SIGMA? At least, the authors should clearly explain why it happens, if it truly should happen.
(6) "for which we defined the conjugate measure G-SIGMA". So instead of clustering cells with SIGMA, here genes are clustered with G-SIGMA? Need better explanation. "Data sets with higher signal-to-noise ratios are characterized by higher values of G-SIGMA (Fig. S6a)". What is signal and what is noise here? "which indicates a more accurate estimation of differential gene expression after subclustering." I totally cannot understand why a higher value of G-SIGMA indicates a more accurate estimation of differential gene expression, if this is what the authors mean? "Furthermore, genes with higher absolute values in a certain gene-singular vector drive the variability observed in the corresponding cell-singular vector (Fig. S6 b-d)." This is too technical and hard to understand. And most critically, are the users supposed to use this information to detect "genes responsible for the detected substructure"? But how exactly do the users do that?
(7) "For both examples, the variance-driving genes found in the gene singular vectors were localized to their corresponding clusters (Fig. S7 c,d) and overlapped strongly with differentially expressed genes found 158 after subclustering (see Supplementary Table 2)." This is not surprising. But more importantly, can't we just identify such variance-driving genes through differential expression analyses of subclusters? There is no need for G-SIGMA then? Which approach is better?
(8) "SIGMA reveals subpopulations in a fetal human kidney data set". All the analyses in this section only support the accuracy of the clustering done by the authors, not the motivation for clustering given by SIGMA. So they do not really give any supporting evidence for SIGMA. In other words, another group of investigators can just perform these clustering practices and draw the same conclusion. There is no need for SIGMA here. This is in contrast to "SIGMA corresponded well with a visual inspection of the cluster UMAPs (Fig. 2a).", where the application in the other dataset does somewhat support the usefulness of SIGMA, though not in the strongest way, either.
Minor comments:
(1) "created new clusters as weighted averages" Meaning is not clear
(2) "As for BMNCs, SIGMA corresponded well with a qualitative assessment of cluster variability and G-SIGMA resulted in a similar ranking (Fig. S8a)." Not sure how this figure supports this claim. The authors need to explain the rationale in the main text.

**Reviewer 3**

The authors propose a method to evaluate the quality of cell clusters obtained by supervised clustering analysis of single cell RNA-seq data. They assume that the matrix of expression data within a cluster is an i.i.d. noise matrix perturbed by a low rank signal matrix. Using some asymptotic results from random matrix theory, they developed a method to estimate the magnitude of the inner products of the top singular vectors between the signal matrix and the data matrix. If these inner products are larger than a certain threshold, then the cluster is judged to have significant sub-structure and may need to be further analyzed. The idea is interesting and the empirical results look promising. However, there are major questions and concerns that should be addressed before we can judge whether this is a useful contribution.
1) What are the advantages, if any, over the more standard approach where one first tries to extract low rank signals and then test whether these signals are significant compared to the estimated additive errors.
2) It is not clear whether the SIGMA statistics is always related to clusterability. In the paper this is demonstrated by just one simulation example. Is it possible to come up with examples whether where there are non-Gaussian clusters that spread out in such as way they captured by a few singular directions.
3) The main use of this method is probably to replace silhouette scores. In figure S5, the silhouette scores seems to offer the same qualitative information as the new method. A more thorough comparison to silhouette scores should be conducted and discussed.

**Authors’ response**

REVIEWER #1

\### 1. L60: low-rank perturbation theory is introduced and referenced but I think it would be helpful to further explain its relevance to random matrix theory and scRNA-seq, particularly for readers not familiar with the method. I would do this by giving an example of the perturbations calculated from an example scRNA-seq dataset, and how this relates to e.g. the singular values obtained from the underlying MP distribution and TW bound. ###

*** Indeed, random matrix theory and low-rank perturbation theory are not so wellknown in the bioinformatics and biology communities. Hence, we heeded the reviewer’s advice and added a more detailed, tutorial-style introduction to the background section. We also created an entire new supplementary figure (new Fig. S1), which illustrates the principles from random matrix theory and low-rank perturbation theory that were applied to derive SIGMA. In the new version of the manuscript, these principles are explained as follows:

“Figure S1 illustrates the basic principles that were applied to derive SIGMA. RMT predicts that the SVD of a random noise matrix results in normal distributed singular vectors and a distribution of singular values that is closely approximated by the MP distribution, if the matrix is large enough (Fig. S1a, left column). Here, we consider the noise-free gene expression profiles of the cells in various phenotypes, as the “signal” that perturbs the random matrix and thus its singular value distribution. Since biological and technical variability are lumped into the random matrix, expression profiles are identical for cells that belong to the same phenotype. For example, in the case of two distinct phenotypes, the signal matrix has only one non-zero singular value (Fig. S1a, middle column). The observed (or measured) gene expression matrix is obtained as the sum of the random noise matrix and the noise-free gene expression profiles (Fig. S1a, right column). The singular value distribution of the measured expression matrix has exactly one singular value above the upper limit that the theory predicts for a purely random matrix, the Tracy-Widom (TW) threshold. The outlying singular value and its associated singular vector correspond to the deterministic component of the measured expression matrix. The distribution of the remaining singular values (the “bulk”) is still closely approximated by the MP distribution. Importantly, as the perturbation becomes larger, the value of the outlying singular value also increases (Fig. S1b). A larger perturbation means more distinct and therefore more easily clusterable phenotypes (compare the singular vectors in the middle row of Figs. S1a and b). The basic idea of SIGMA is to use the magnitude of the outlying singular values to quantify clusterability. Due to the universality of RMT, all described principles are independent of the particular noise distribution (see Fig. S1a-b for normal distributed noise and Fig. S1c-d for Poisson distributed noise). SVD of appropriately preprocessed real data sets therefore leads to singular value distributions with the same shape as obtained in simulations: a bulk closely approximated by the MP distribution and one or multiple outlying values.”

As specifically requested by the reviewer, we now also show, how a perturbation relates to the singular value distribution of an experimental scRNA-seq measurement (Fig. S2f,g). Since it is not possible to reconstruct the noise-free perturbation from a noisy data set, we rather added an additional perturbation to the measured expression matrix. In this way we preserve the noise distribution of real data and can track, how the additional perturbation manifests itself in the singular value distribution. We added the following description to the manuscript:

“We next wanted to confirm, for real data, that the remaining outlying singular values reflect the strength of the signal, i.e., differences between the phenotypes. To that end, we extracted the gene expression profiles from two clusters in an experimental singlecell RNA-seq data set and added, as additional signal, a matrix with one non-zero singular value. As to be expected, SVD of the combined data results in one additional singular value, which increases with the strength of the perturbation (S2f-g). See Supplementary Table 2 for a list of all outlying singular values of experimentally measured expression matrices as well as the corresponding signal matrices. All in all, these tests show that the basic principles of random matrix theory and perturbation theory are applicable to real single-cell RNA-seq data.” ***

\### 2. L97: It is not clear how the Bayesian error rate corresponds to the lowest possible error in this application. Can you further explain and provide an example? ###

*** In the new version of the manuscript, we have significantly extended the explanation of the Bayes error rate and improved the graphical representation of an example (Fig. S3). We hope that these improvements satisfy the reviewer’s request.

The new paragraph in the manuscript reads as follows:

“Take, for instance, the simplest possible case of two cell types, A and B. Without any noise (technical or biological), expression profiles within a cell type are identical and the data can be clustered perfectly. Correspondingly, the singular vector of the expression matrix has only two different entries (Fig. S3a, left). Therefore, it is easy to find a threshold that discriminates between the two cell types. In the presence of noise, however, there is a chance that the measured expression profile of a cell from cell type A looks more like cell type B and is therefore clustered with other cells from cell type B and vice versa. Correspondingly, the entries of the singular vector are now spread by the noise and can overlap (Fig. S3a, right). Even if we use the best possible threshold to discriminate between the two cell types, some cells will be necessarily misclassified, if the distributions overlap. This type of error is unavoidable (or irreducible) and known as Bayes error rate [23] in the context of statistical classification. From the overlap of the singular vector entries, we can calculate the Bayes error rate or, equivalently, a theoretically achievable ARI (tARI, see also Supplementary Note 1). Of course, this is only possible for data with known ground truth. We first used simulated data to show empirically that commonly used clustering methods are not able to exceed the tARI (Fig. S3b,c). It therefore quantifies our notion of clusterability: With increased noise, tARI decreases and it is more challenging even for the best conceivable clustering algorithm to distinguish the difference between phenotypes. Importantly, SIGMA is strongly correlated with the tARI (Fig. 1d) and thus allows us to estimate clusterability without knowing the ground truth. “ ***

\### 3. L122: The need to regress out nuisance parameters is a vital step in the SIGMA calculation, and as such I feel there should be a more extensive discussion of this step in the main text. I propose you move the Supplementary Figure 4, where the effects of the regression are shown, to the main body and describe it there. I would also add a table where the differences in the SIGMA value before and after correcting for the confounding nuisance parameters are stated for each data-set. It would also be interesting to comment on the relevant sizes of different nuisance parameters to the data (see comment 5). ###

*** We completely agree that the confounder regression is critical for our method. Following the reviewer’s advice, we moved the relevant figures from the supplementary figure to the main figures (new Fig. 1f,g and Fig. 2c,d) and added a table that lists the size of all nuisance parameters, as well as the corrected and uncorrected singular values and values of SIGMA (new supplementary table 2). We also describe the regression procedure in more detail in the main text and the methods section:

“Confounder regression removes unwanted variability To further characterize the performance of SIGMA on experimental data sets with known ground truth, we used a measurement of purified RNA from 3 cell types, mixed at different ratios [25] (Fig. 1e). We noticed a significant correlation between the amount of input RNA and the entries of the first singular vector of individual clusters (Fig. 1f). This might be explained by lowly expressed genes not being well-represented in the low-input libraries, and the resulting differences in the expression profiles. In any case, the amount of input RNA seemed to be a confounding factor that could lead to high values of SIGMA, even in the absence of meaningful subclusters.

Correspondingly, we found a correlation between the singular vector entries and the number of total counts, despite normalization of the data (Fig. 1g). This is consistent with the finding that total counts are a confounding factor in scRNA-seq data that cannot be eliminated by normalization using one single scaling factor per cell [24,26]. Different groups of genes scale differently with the total counts per cell. Therefore, a correlation with the total counts remains even after normalization. More generally, there are various experimental and biological factors that drive artefactual or irrelevant variability in single-cell RNA-seq data [24,27]. We therefore introduced a regression step that removes the influence of any nuisance variables, such as the number of total counts per cell, ribosomal gene expression, mitochondrial gene expression or cell cycle phase (see also Supplementary Note 1). More specifically, we first regress the entries of a singular vector on one or multiple confounders. The fraction of variance explained by all confounder is then given by the adjusted R2 (coefficient of determination) of the linear regression. Since the squared singular values can also be interpreted as the amount of variance explained, we correct them by multiplying with 1 – the adjusted R2 found in the confounder regression. (See Supplementary Table 2 for a list of the uncorrected and corrected singular values for both simulated and experimental expression matrices.) The corrected singular values are then used to calculate SIGMA.”

In the new figure S5b we show the (substantial) effect of confounder regression and subsequent correction of SIGMA graphically:

“In Supplementary Table 2 we list the R2 values of each considered confounder for each cluster. For real scRNA-seq data sets, confounder regression can lead to a significant reduction of SIGMA (Fig. S5b, see Supplementary Table 2 for the numerical values.) It is therefore an important part of the method. “ ***

\### 4. L188-191: The choice of random matrix models assumes the noise is additive, which is justified given the results in this study with SIGMA. It would be helpful to quantify how good this assumption is by including a table of the closeness of singular values to the MP distribution, and indicate how much this changes between different data-sets. This way, it will be possible to make a rigorous statement about how much the MP behaviour varies between the different data-sets, and to confirm that the additive assumption of noise is valid for all data-sets in the study. Given the closeness of the behaviour to the MP distribution, it might also be possible to derive a modelling uncertainty on SIGMA. I think this would be useful as it can be directly translated into essentially a confidence interval on the results of the SIGMA clustering quality, where the size of the interval depends on the closeness of the MP modelling assumption to the truth. ###

*** We are grateful to the reviewer for this remark, because it prompted us to improve our manuscript in multiple ways. We realized that the previous version of the manuscript contained a paragraph that was imprecise and probably misleading for the reader. Previously we wrote in the discussion: “… but it has to be assumed that the noise is additive. Under this assumption, random matrix theory predicts the bulk of the singular value distribution to follow the Marchenko-Pastur distribution. Empirically, we find this to be true to a high degree of approximation, which supports the assumption of additive noise. ” In fact, the bulk of the singular value distribution will always be approximated by the MP distribution, no matter if the noise is additive or multiplicative. We apologize for the confusion the old phrasing may have caused. We have now also calculated SIGMA for multiplicative noise and added this paragraph to the manuscript: “So far, we have assumed additive noise (i.e., the measured gene expression is the sum of a random matrix and the noise-free expression matrix). Low-rank perturbation theory also makes a prediction for multiplicative noise (i.e., the measured gene expression is the product of a random matrix and the noise-free expression matrix). In that case, SIGMA still scales approximately linearly with the tARI, but its dynamic range depends somewhat on the cell-to-gene ratio (Fig. S3d). To our knowledge, the noise generating mechanisms at work in scRNA-seq have not been pinpointed comprehensively. Therefore, we will continue to assume additive noise, noting that our approach can be easily adapted to multiplicative noise.”

Nevertheless, we assessed the closeness of the singular value distribution bulk to the MP distribution with a Kolmogorov-Smirnov test and found no significant deviation, with the exception of one cluster in the BMNC data set (Fig. S2e):

“We tested for all data sets used in this study, whether the bulk of the singular value distribution of each cluster deviates significantly from the MP distribution after the described preprocessing (Kolmogorov-Smirnov test, Fig. S2e). For reasonably large clusters (> 50 cells), we only found one example of a (marginally significant) deviation from the MP distribution.”

This result supports the assumption, that the noise component of scRNA-seq data can be understood as a sampling from independent and identically distributed random variables. The shape of the bulk of the singular value distribution is independent of the type of noise and also any deterministic signal in the data. Therefore, a goodness-of-fit comparison to the MP distribution can unfortunately not be used to calculate the uncertainty for SIGMA, which is derived from the outlying singular values (i.e., the deterministic component of the data). We do agree with the reviewer that it is very useful to estimate the uncertainty of SIGMA. Hence, we devised the following estimation procedure described in the Methods section:

“Uncertainty of SIGMA

The uncertainties for the values of SIGMA are estimated using a sampling approach. The basic idea is to approximate the signal matrix P and add new realizations of the noise matrix by sampling from a random distribution. The uncertainty is then obtained from the values of SIGMA calculated for this ensemble of sampled matrices. First, we decompose a simulated or measured expression matrix X ̃ into a noise matrix Xr and a matrix Xs that contains deterministic structure. Xs is constructed from the relevant singular vectors, which were identified as described in the previous section. Note that Xs contains noise and is thus different from the signal matrix P. To create an approximation Ps of the signal matrix P, we replace the singular values γ_i used in the construction of Xs with the singular values θ_i of P, calculated using low-rank perturbation theory as shown in the previous section. The entries of the noise matrix Xr have a mean of 0 and a standard deviation of 1, as a result of preprocessing. Since the results of RMT are valid irrespective of the particular noise distribution, we can create additional realizations of the noise matrix by sampling from a normal distribution with mean 0 and standard deviation 1. By adding sampled noise matrices to the approximated signal matrix Ps, we can create an ensemble of matrices with the same singular value spectrum as the original measured expression matrix but different realizations of the noise. The uncertainty for positive and negative deviations from the mean is then calculated as the standard deviation for at least 50 sampled matrices. See Supplementary Note, Section 2.4.3 for a detailed description.” ***

\### 5. L194: Does the hierarchy of the size of different confounding factors/nuisance parameters (RNA recovery vs. cell cycle phase dependence) vary between different data-sets, or is one effect dominating across most data-sets? If so, it would be interesting to quantify this effect and provide a possible explanation for why it is observed. A table of the various contributions of different nuisance parameters would be a welcome addition. ###

*** For the data sets we analysed, there is no single factor that is clearly dominating across all data sets. The relative size of various confounders is rather data setdependent. In the new figure S5a we report the relative impact of the nuisance parameters and we discuss possible reasons for the observed differences in the text:

“Interestingly, the relative influence of the confounders considered in this study varied substantially between data sets (Fig. S5a). For example, cell stress is a relevant confounder only in the kidney data set. This is likely related to the cell dissociation procedure, which is necessarily more aggressive for kidney tissue, compared to the other samples: bone marrow mononuclear cells (BMNCs) and purified RNA, extracted from cell cultures. Total counts and ribosomal gene expression explain most of the artefactual variance in BMNCs. This might be explained by high variability in the metabolic state of the cells. “

As already mentioned in the response to remark 3, the new supplementary table 2 contains a list of the coefficients of determination for all considered nuisance parameters in all data sets, as well as uncorrected and corrected numerical values for the outlying singular values and SIGMA itself. ***

REVIEWER #2

Major comments:

\### (1) Several places in the introduction need to be clarified to make sure readers understand the key logic flow of the story. ###

*** We very much appreciate the reviewer for pointing out the technical nature of our manuscript which might make it hard for a reader without an extensive background in the employed theories to follow our key arguments. To address the reviewer’s point, we have significantly extended the background section with an explanation of the rationale and most important results of random matrix theory:

“Below we will use results of RMT on the singular value decomposition (SVD) of a single-cell gene expression matrix, where rows correspond to genes and columns correspond to cells. To get an intuitive understanding of RMT, it is useful to first consider the cell-cell correlation matrix, calculated from the gene expression profiles. We start from the null hypothesis that the data does not contain any structure and is produced by a random process. In the context of single-cell transcriptomics, “structure” means multiple, distinguishable clusters of cells, or phenotypes. RMT can predict, what the correlation matrix looks like, if the entries of the gene expression matrix are samples of random variables that are independent and identically distributed. Trivially, the diagonal elements of this correlation matrix are all equal to 1. The off-diagonal elements are not exactly 0, however, despite the absence of any meaningful structure in the data. Only in the limit of measuring an infinite number of (random) genes would the off-diagonal elements become identically 0 and the correlation matrix would become the identity matrix. In that case, the only eigenvalue of the correlation matrix is 1. RMT describes the properties of a correlation matrix for a finite ratio of cells and genes. These correlation matrices are, in a sense, distributed “around” the identity matrix, which corresponds to an eigenvalue spectrum distributed around 1. Although the individual entries of the correlation matrix fluctuate from realization to realization, RMT shows that the eigenvalue spectrum is robust (a so-called “self-averaging” property) and an analytical expression for it can be obtained [14]. Likewise, RMT predicts that the singular value distribution of a purely random matrix is closely approximated by the Marchenko-Pastur (MP) distribution. This result holds true irrespective of the distribution of the random variable. This universal property of random matrices allows us to apply RMT to gene expression matrices obtained by scRNA-seq. Of course, any biologically interesting scRNA-seq measurement should contain structure, usually in the form of cell clusters. RMT allows us to regard singular values lying above the MP distribution as evidence for the rejection of the null hypothesis (i.e., the absence of structure in the data). The MP distribution is characterized by sharp upper and lower limits for the singular values of a random matrix, but is strictly valid only in the limit of infinite numbers of genes and cells (while keeping the cell-gene ratio fixed). For finite matrices, the largest and smallest singular values are distributed around those sharp limits, which is described by the Tracy- Widom distribution [15].

As explained above, the presence of structure manifests itself as singular values above the MP distribution (i.e., the prediction for a purely random matrix). Qualitatively, the magnitude of those outlying singular values corresponds to the magnitude of the differences between clusters. We can understand this relationship, if we assume that the measured gene expression matrix is the sum of a random matrix (the “noise”) and a matrix of noise-free gene expression profiles (the “signal”), see Fig. 1a. The bigger the difference in gene expression between phenotypes, the larger the magnitude of the non-zero singular values of the signal matrix. If the number of non-zero singular values (i.e., the rank of the signal matrix) is small compared to the dimensions of the matrix, low-rank perturbation theory [16] is applicable. This theory allows us to calculate the singular values of the measured gene expression matrix from the singular values of the signal matrix. Remarkably, knowledge of the complete signal matrix is not required for this calculation. The theory also predicts the angle between corresponding singular vectors in the signal and noise matrix. If the signal is strong (i.e., the non-zero singular values of the signal matrix are high) compared to the noise, corresponding singular vectors will have a small angle: The measured expression matrix will look very similar to the signal. If the signal is weak, the correlation between the signal and the measured expression will go to 0, which corresponds to orthogonal singular vectors. This is just a consequence of the law of large numbers: two random vectors of unit length will have 0 correlation (and therefore scalar product 0) in the infinite limit. The smallest angle thus corresponds to the strongest signal. We will define the squared cosine of the smallest angle as our measure SIGMA.“ ***

\### (a) "Notwithstanding its usefulness, it cannot be excluded that a partition of random noise obtains a high silhouette coefficient, indicating high clustering quality." Doesn't SIGMA suffer from the same problem? ###

*** Fortunately, SIGMA does not suffer from this problem. A cluster consisting only of random noise, as well as all possible sub-clusters of it, receive a SIGMA value of identically 0, which indicates that there is no reason to sub-cluster. We now mention this fact explicitly in the manuscript:

“If SIGMA equals 0, the observed variability within a cluster is consistent with random noise. Any subclusters of such a cluster still have a SIGMA of 0, which prevents over-clustering of random noise.”

We also added a new supplementary figure (Fig. S7) to compare SIGMA to the silhouette coefficient in more detail. As discussed in a new section of the manuscript, using the silhouette coefficient can lead to over-clustering of noise, while SIGMA correctly indicates the absence of any meaningful structure:

“To compare SIGMA with the silhouette coefficient in more detail, we carried out additional simulations (Fig. S7). First, we simulated 3 clusters and subsequently merged two of them. While SIGMA clearly distinguished the merged cluster from the pure cluster, the silhouette coefficients were similar for both. Increasing the fraction of genes that are differentially expressed between the merged cluster increased the difference in silhouette coefficient, but only gradually (Fig. S7b). By contrast, SIGMA jumped to values close to 1 for the merged cluster for very small fractions of differentially expressed genes (around 0.03). It is therefore the more sensitive measure. The silhouette coefficient strongly depends on the number of principal components used in dimensionality reduction (Fig. S7c), as well as the metric for distances between expression profiles (Fig. S7d). SIGMA does not depend on such user-defined parameters. Most importantly, the silhouette coefficient cannot answer the question, whether an identified cluster contains meaningful substructure, as it requires partitioning into at least 2 sub-clusters. We simulated a cluster without any substructure and all variability was purely random (Fig. S7e). The silhouette coefficient was maximal for a k-means clustering with k=2, which might prompt a user to conclude (wrongly) that there are 2 sub-clusters present. SIGMA, which does not require any further partitioning of the cluster, was 0, indicating correctly that the observed variability was consistent with random noise.” ***

\### (b) "We consider clusterability to be the theoretically achievable agreement with the unknown ground truth clustering, for a given signal-to-noise ratio." It will be important to introduce what is signal and what is noise here. ###

*** We agree with the reviewer that a better definition was in order. We added the following paragraph to the manuscript:

“We can understand this relationship, if we assume that the measured gene expression matrix is the sum of a random matrix (the “noise”) and a matrix of noise-free gene expression profiles (the “signal”), see Fig. 1a. The bigger the difference in gene expression between phenotypes, the larger the magnitude of the non-zero singular values of the signal matrix.” ***

\### (c) "Notions of clusterability have been proposed previously [6,9], but those are not directly compatible with scRNA-seq data". Need to state what exactly are the notions that have been proposed and why they are not compatible. Simply citing papers is not enough here. ###

*** Our explanation was indeed too brief. We now explicitly state the shortcomings of existing methods:

“To assess the quality of a clustering quantitatively and help choose optimal parameters, some measures of clustering quality and clusterability have been proposed [6,7], most of which are not directly applicable to scRNA-seq data. For example, some existing methods rely on multimodality of the expression matrix, which is not always justified for scRNA-seq data, especially when considering highly dynamic systems. Other methods have input parameters, such as the optimal number of dimensions for dimensionality reduction, that cannot be easily determined. Also, general methods do not explicitly account for uninformative sources of variability, related to cell cycle progression or the stress response, for example, which can be important confounders.” ***

\### (2) Same as above. "This is made possible by using universality properties of random matrix theory." Need some intuitive introduction on the random matrix theory and how it is used here. ###

***For our method, the most important universal property of a random matrix is its singular value distribution. Irrespective of the particular random process and the associated noise distribution, the singular value distribution is always the Marchenko- Pastur distribution. For real data, the bulk of the singular value distribution is closely approximated by the Marchenko-Pastur distribution. The universal nature of this distribution is now mentioned explicitly in the text:

“Likewise, RMT predicts that the singular value distribution of a purely random matrix is closely approximated by the Marchenko-Pastur (MP) distribution. This result holds true irrespective of the distribution of the random variable. This universal property of random matrices allows us to apply RMT to gene expression matrices obtained by scRNA-seq.”***

\### "We expand on these results by applying low-rank perturbation theory [13], which allows us to identify the signature of deterministic signals in the context of random noise." Same for "low-rank perturbation theory". ###

***We agree that more context was needed. We tried to express the main results and use of perturbation theory in this new paragraph of the manuscript:

“The bigger the difference in gene expression between phenotypes, the larger the magnitude of the non-zero singular values of the signal matrix. If the number of nonzero singular values (i.e., the rank of the signal matrix) is small compared to the dimensions of the matrix, low-rank perturbation theory [16] is applicable. This theory allows us to calculate the singular values of the measured gene expression matrix from the singular values of the signal matrix. Remarkably, knowledge of the complete signal matrix is not required for this calculation. The theory also predicts the angle between corresponding singular vectors in the signal and noise matrix. If the signal is strong (i.e., the non-zero singular values of the signal matrix are high) compared to the noise, corresponding singular vectors will have a small angle: The measured expression matrix will look very similar to the signal. If the signal is weak, the correlation between the signal and the measured expression will go to 0, which corresponds to orthogonal singular vectors. This is just a consequence of the law of large numbers: two random vectors of unit length will have 0 correlation (and therefore scalar product 0) in the infinite limit. The smallest angle thus corresponds to the strongest signal. We will define the squared cosine of the smallest angle as our measure SIGMA.”

As specifically requested by reviewer #1, we now also show, how a perturbation relates to the singular value distribution of an experimental scRNA-seq measurement (Fig. S2f,g). Since it is not possible to reconstruct the noise-free perturbation from a noisy data set, we rather added an additional perturbation to the measured expression matrix. In this way we preserve the noise distribution of real data and can track, how the additional perturbation manifests itself in the singular value distribution. We added the following description to the manuscript:

“We next wanted to confirm, for real data, that the remaining outlying singular values reflect the strength of the signal, i.e., differences between the phenotypes. To that end, we extracted the gene expression profiles from two clusters in an experimental singlecell RNA-seq data set and added, as additional signal, a matrix with one non-zero singular value. As to be expected, SVD of the combined data results in one additional singular value, which increases with the strength of the perturbation (S2f-g). See Supplementary Table 2 for a list of all outlying singular values of experimentally measured expression matrices as well as the corresponding signal matrices. All in all, these tests show that the basic principles of random matrix theory and perturbation theory are applicable to real single-cell RNA-seq data.”***

\### And what do you mean by "deterministic signals"? ###

***We mean everything that is not modeled by a random process. Here, it corresponds to the meaningful structure in the data, the actual cell clusters. We use “non-random” and “deterministic” synonymously in the manuscript:

“SIGMA is meant to help identify non-random (or deterministic) structure.”***

\### "SIGMA is derived from first principles and does not have free parameters". What are "first principles" and "free parameters"? ###

***We consider first principles to be axioms or assumptions that either don’t require further justification or cannot be justified by even more fundamental assumptions. They are the best possible starting point for a rigorous mathematical derivation. To analyze the singular value distribution of the measured expression matrices we only use general (or universal) properties of random matrices and perturbation theory. Most importantly, our model is not informed by the data. An empirical or heuristic approach, which starts with the data, can thus be considered the opposite of arguing from first principles. We believe that a principled approach is more general and less biased than an empirical approach, whose results might depend sensitively on the choice of data sets used to derive it. We now make an explicit remark about these differences in the manuscript:

“We would like to stress at this point that SIGMA is derived from universal properties of perturbed random matrices, which can be considered first principles. By contrast, many other measures are developed based on empirical observations and justified post hoc by their usefulness. SIGMA is calculated using only the SVD and the dimensions of the expression matrix. Thus, it does not have any free, adjustable parameters, which would have to be chosen by the user or learned from the data.”***

\### (3) In Fig. S1, what is "transcriptome mode"? ###

***The transcriptome mode corresponds to a global trend in the gene expression profiles that can be found across all phenotypes in a data set. If it is present in a data set, it is typically the singular vector with the largest singular value and this singular value is much larger than all other singular values. Also, all entries of this singular vector have the same sign and similar magnitude. The transcriptome mode is therefore not informative for clustering and can be removed by scaling the data. We added a more detailed explanation to the manuscript:

“SVD of raw data or log-transformed, normalized data typically results in a largest outlying singular value that is much larger than the rest (Fig. S2a,b). The corresponding singular vector reflects a global trend in the data and is called “market mode” in the context of stock market analysis [10,18]. Here, we call it “transcriptome mode”, since it corresponds to an expression trend that is present across all cells, irrespective of cell type (such as, for example, high expression of cytoskeletal genes and low expression of membrane receptors). The transcriptome mode is obviously not informative for clustering. Scaling shifts its singular value to 0, which effectively removes it from further analysis (S2c,d).”***

\### "If the data is preprocessed appropriately (Fig. S1)," After reading Fig. S1, I still don't quite get what constitutes "appropriate preprocessing". ###

***We found that preprocessing should comprise normalization, log-transformation and scaling, both gene- and cell-wise. After these steps, all clusters in the studied data sets had singular value distributions whose bulk was closely approximated by the Marchenko-Pastur distribution, with the exception of one cluster, which showed a marginally significant deviation. We added this explanation to the manuscript:

“We found that data preprocessing has to comprise normalization and logtransformation, as well as gene-wise and cell-wise scaling (Fig. S2a-d). … We tested for all data sets used in this study, whether the bulk of the singular value distribution of each cluster deviates significantly from the MP distribution after the described preprocessing (Kolmogorov-Smirnov test, Fig. S2e). For reasonably large clusters (> 50 cells), we only found one example of a (marginally significant) deviation from the MP distribution. ”***

\### "The bulk of this distribution is described by the Marchenko-Pastur (MP) distribution, which corresponds to the random component of the measurement" Why? ###

***This is an important but also difficult question. The fact that the bulk of the distribution approximates the MP distribution is a non-trivial result from random matrix theory. Its proof involves advanced mathematics and we feel that it would require too much space to rigorously derive the relevant results in this manuscript. We would also like to refer the reviewer to Supplementary Note 1, which describes many of the used concepts in a bit more detail. We have tried to give some intuitive arguments in the new background section:

“To get an intuitive understanding of RMT, it is useful to first consider the cell-cell correlation matrix, calculated from the gene expression profiles. We start from the null hypothesis that the data does not contain any structure and is produced by a random process. In the context of single-cell transcriptomics, “structure” means multiple, distinguishable clusters of cells, or phenotypes. RMT can predict, what the correlation matrix looks like, if the entries of the gene expression matrix are samples of random variables that are independent and identically distributed. Trivially, the diagonal elements of this correlation matrix are all equal to 1. The off-diagonal elements are not exactly 0, however, despite the absence of any meaningful structure in the data. Only in the limit of measuring an infinite number of (random) genes would the off-diagonal elements become identically 0 and the correlation matrix would become the identity matrix. In that case, the only eigenvalue of the correlation matrix is 1. RMT describes the properties of a correlation matrix for a finite ratio of cells and genes. These correlation matrices are, in a sense, distributed “around” the identity matrix, which corresponds to an eigenvalue spectrum distributed around 1. Although the individual entries of the correlation matrix fluctuate from realization to realization, RMT shows that the eigenvalue spectrum is robust (a so-called “self-averaging” property) and an analytical expression for it can be obtained [14]. Likewise, RMT predicts that the singular value distribution of a purely random matrix is closely approximated by the Marchenko-Pastur (MP) distribution.***

\### "we can calculate the angles between the singular vectors of the measured gene expression matrix and those of the (unobserved) signal matrix" How is this possible without knowing the unobserved signal matrix? ###

***The result from low-rank perturbation theory the reviewer refers to were obtained using a non-trivial mathematical analysis of perturbed random matrices [1] using advanced concepts from free probability theory. It is therefore difficult to find an intuitive explanation that is also accurate. We can roughly rationalize the result like this:

The law of large numbers dictates that two completely random vectors will have a scalar product of zero in the infinite limit. If two vectors are identical (and of unit length), the scalar product is 1. The scalar product of the corresponding singular vectors of signal and noise will generally lie between these extremes. Weak signals will be randomized by noise, so the scalar product will be closer to 0. Strong signals will not be easily degraded by random noise and the scalar product will be closer to 1. Note that we only needed the relative strengths of signal and noise (i.e., the magnitudes of the singular values) and not the expression profiles themselves to make the preceding arguments. Unfortunately, some of the results of perturbation theory are in fact quite unintuitive. For example, the theory predicts that, if the largest singular value of the signal matrix is below a certain threshold, there is no significant (or outlying) singular value in the perturbed matrix. This “phase transition” behavior can be observed in Fig. 1c as SIGMA being identically 0 initially. While unexpected and unintuitive, we could confirm this result empirically for all data sets with known ground truth. In the new version of the manuscript, we added the following paragraph to add as much intuition as possible:

“The theory also predicts the angle between corresponding singular vectors in the signal and noise matrix. If the signal is strong (i.e., the non-zero singular values of the signal matrix are high) compared to the noise, corresponding singular vectors will have a small angle: The measured expression matrix will look very similar to the signal. If the signal is weak, the correlation between the signal and the measured expression will go to 0, which corresponds to orthogonal singular vectors. This is just a consequence of the law of large numbers: two random vectors of unit length will have 0 correlation (and therefore scalar product 0) in the infinite limit. The smallest angle thus corresponds to the strongest signal. We will define the squared cosine of the smallest angle as our measure SIGMA.”***

\### Some intuition and laymen language need to be put into the main text, rather than stacked technical jargons. ###

***We followed the reviewer’s advice and hope that the new version is more understandable.***

\### (4) The same problem here: "Using a simple case of two clusters of cells with varying signal-to-noise ratios, we estimated the Bayesian error rate [18] (i.e. the lowest possible error) for this binary classification problem in simulated data (Fig. S2a). Based on this error rate, we calculated a theoretically achievable ARI (tARI, see also Supplementary Note 1). We showed empirically that commonly used clustering methods do not exceed this limit (Fig. S2b,c)." The authors need to point out intuitively what has been done, instead of simply pointing to a figure or supplemental material. For example, what is signal and what is noise in this sentence: "varying signal-to-noise ratios". After reading this part, I am totally unsure what was done and whether it is correct or not. ###

***The definitions of “signal” and “noise” are as defined before. We vary the strength of the signal, by changing the amount of differentially expressed genes or the fold-change of differential expression. This new paragraph in the manuscript describes the construction of the tARI in more detail:

“Take, for instance, the simplest possible case of two cell types, A and B. Without any noise (technical or biological), expression profiles within a cell type are identical and the data can be clustered perfectly. Correspondingly, the singular vector of the expression matrix has only two different entries (Fig. S3a, left). Therefore, it is easy to find a threshold that discriminates between the two cell types. In the presence of noise, however, there is a chance that the measured expression profile of a cell from cell type A looks more like cell type B and is therefore clustered with other cells from cell type B and vice versa. Correspondingly, the entries of the singular vector are now spread by the noise and can overlap (Fig. S3a, right). Even if we use the best possible threshold to discriminate between the two cell types, some cells will be necessarily misclassified, if the distributions overlap.

This type of error is unavoidable (or irreducible) and known as Bayes error rate [23] in the context of statistical classification. From the overlap of the singular vector entries, we can calculate the Bayes error rate or, equivalently, a theoretically achievable ARI (tARI, see also Supplementary Note 1). Of course, this is only possible for data with known ground truth. We first used simulated data to show empirically that commonly used clustering methods are not able to exceed the tARI (Fig. S3b,c). It therefore quantifies our notion of clusterability: With increased noise, tARI decreases and it is more challenging even for the best conceivable clustering algorithm to distinguish the difference between phenotypes. Importantly, SIGMA is strongly correlated with the tARI (Fig. 1d) and thus allows us to estimate clusterability without knowing the ground truth. “***

\### (5) "We noticed that the amount of input RNA used for each mixture was a confounding factor that influenced the value of SIGMA (Fig. S4b,c)." This seems to be a critical problem affecting SIGMA. Why would the amount of input RNA affect clustering results? Usually, the expression counts of each cell were normalized to something like TPM, and theorectically, the amount of input RNA will already be irrelevant after this step, and should not affect SIGMA? At least, the authors should clearly explain why it happens, if it truly should happen. ###

***The correlation with the total counts per cell even after normalization with a single factor per cell (such as in TPM normalization) is an established phenomenon that is explicitly addressed in the latest normalization methods (e.g., sctransform) or dimensionality reduction methods (e.g., ZINB-WaVE). The main reason is that different groups of genes scale differently with the total counts (which depend strongly on the RNA input in the case of the RNA mixture data set). We explained the problem in more detail in the new version of the manuscript:

“We noticed a significant correlation between the amount of input RNA and the entries of the first singular vector of individual clusters (Fig. 1f). This might be explained by lowly expressed genes not being well-represented in the low-input libraries, and the resulting differences in the expression profiles. In any case, the amount of input RNA seemed to be a confounding factor that could lead to high values of SIGMA, even in the absence of meaningful subclusters. Correspondingly, we found a correlation between the singular vector entries and the number of total counts, despite normalization of the data (Fig. 1g). This is consistent with the finding that total counts are a confounding factor in scRNA-seq data that cannot be eliminated by normalization using one single scaling factor per cell [24,26]. Different groups of genes scale differently with the total counts per cell. Therefore, a correlation with the total counts remains even after normalization.”***

\### (6) "for which we defined the conjugate measure G-SIGMA". So instead of clustering cells with SIGMA, here genes are clustered with G-SIGMA? Need better explanation. ###

***We hope that the extended background section in the new version of the manuscript has made it clearer, that SIGMA is not a clustering algorithm. It rather provides a quantitative assessment of an existing cluster. A SIGMA value close to 1 indicates a cluster with additional structure that should be easily distinguishable from random noise. In other words, a cluster with a high value of SIGMA can and should be subclustered to identify additional, subtle phenotypes. Similarly, G-SIGMA assesses an existing cluster, but it makes a statement about gene space. Larger values of GSIGMA indicate, that meaningful gene expression variability (such as differences between multiple phenotypes) are more easily distinguishable from noise. The user will thus have more significant genes to work with for the biological interpretation. We have added a more detailed description of G-SIGMA to the manuscript:

“In full analogy to the reasoning outlined so far, our approach can also be used to characterize variability in gene space, for which we defined the conjugate measure GSIGMA (see Supplementary Note 1 for the derivation). Above, we considered only the right singular vectors, where each entry corresponds to a cell in the data set. We therefore also call them “cell-singular vectors”. In the simplest case of well separated clusters, entries in the cell singular vectors indicate the membership of a cell in a cluster or a group of clusters. For the left singular vectors, each entry corresponds to a gene. Therefore, we also call them “gene-singular vector”. The squared cosine of the angle between the leading gene-singular vector in the measured gene expression matrix and the corresponding gene-singular vector of the noise-free signal matrix is GSIGMA. As for SIGMA, data sets with higher signal-to-noise ratios are characterized by higher values of G-SIGMA (Fig. S8a). “Signal” and “noise” are defined exactly as above: “noise” is a random matrix and the “signal” is a low-rank matrix consisting of noise-free expression profiles, where the strength of the signal (or difference between the clusters) corresponds to the magnitude of the non-zero singular values. A GSIGMA close to 0 would indicate that all observed differential gene expression can be explained by random noise. Larger values of G-SIGMA indicate less overlap of the gene expression profiles between phenotypes. We therefore expect to find a bigger number of significantly differentially expressed (DE) genes and/or larger fold changes between phenotypes.”***

\### "Data sets with higher signal-to-noise ratios are characterized by higher values of G-SIGMA (Fig. S6a)". What is signal and what is noise here? ###

***The definitions of signal and noise remain the same throughout the manuscript. We now mention that explicitly in the text:

“ “Signal” and “noise” are defined exactly as above: “noise” is a random matrix and the “signal” is a low-rank matrix consisting of noise-free expression profiles, where the strength of the signal (or difference between the clusters) corresponds to the magnitude of the non-zero singular values.”***

\### "which indicates a more accurate estimation of differential gene expression after subclustering." I totally cannot understand why a higher value of G-SIGMA indicates a more accurate estimation of differential gene expression, if this is what the authors mean? ###

***As already mentioned above, G-SIGMA is the exact equivalent of SIGMA in gene space. While SIGMA quantifies the strength of the signal or structure with respect to noise in cell space, G-SIGMA does the same in gene space. In the simplest case of two sub-populations within a cluster, a high value of G-SIGMA signifies more distinct gene expression differences between the two sub-populations. These differences can come from higher fold-changes (compared to the noise level) or more genes that are different between the sub-populations. For the revised version of the manuscript, we rephrased our explanation of the meaning of G-SIGMA in the following way:

“Larger values of G-SIGMA indicate less overlap of the gene expression profiles between phenotypes. We therefore expect to find a bigger number of significantly differentially expressed (DE) genes and/or larger fold changes between phenotypes.”***

\### "Furthermore, genes with higher absolute values in a certain gene-singular vector drive the variability observed in the corresponding cell-singular vector (Fig. S6 b-d)." This is too technical and hard to understand. And most critically, are the users supposed to use this information to detect "genes responsible for the detected substructure"? But how exactly do the users do that? ###

***To interpret the results of SIGMA and assess, whether it is worthwhile to sub-cluster an existing cluster and validate the resulting sub-clusters, the user can use what we define as “variance-driving genes”. Those are the genes with the highest entries in the gene-singular vectors with outlying singular values. The bigger the magnitude of an entry, the larger the variability of this gene within the cluster and the larger the difference between potential sub-clusters. By studying the list of genes with the largest entries, the user can achieve a preliminary biological interpretation of the potential substructure. We have tried to explain this procedure in a new paragraph in the revised manuscript:

“We confirmed by simulations that genes with larger absolute entries in a genesingular vector contribute more to the differences between the clusters separated along the corresponding cell-singular vector (Fig. S8b-d): For example, if two clusters (A and B) are separated along a cell-singular vector and cells in cluster A are characterized by positive entries, the genes with large positive entries in the corresponding gene-singular vector will be mostly expressed in cluster A. We call these “variance driving” genes.” ***

\### (7) "For both examples, the variance-driving genes found in the gene singular vectors were localized to their corresponding clusters (Fig. S7 c,d) and overlapped strongly with differentially expressed genes found 158 after subclustering (see Supplementary Table 2)." This is not surprising. But more importantly, can't we just identify such variance-driving genes through differential expression analyses of subclusters? There is no need for G-SIGMA then? Which approach is better? ###

***We believe that finding variance-driving genes and the differential gene expression (DGE) are complementary. Importantly, variance-driving genes can be identified without sub-clustering of the data and no assumptions about the distribution of expression data are necessary. DGE might be biased by a particular sub-clustering and typically assumes certain distributions for single-cell gene expression. On the other hand, DGE can identify statistical significance. In the revised version of the manuscript, we highlight the advantages of using variance-driving genes in this new paragraph:

“In contrast to differential expression tests, the variance driving genes can be obtained before clustering and might help the user interpret the observed variability and make an informed decision on whether it is useful to sub-cluster the data. If the variance driving genes have enriched biological features (such as being involved in the same signaling pathway or cellular function), we can take that as additional evidence for biologically meaningful sub-population.”***

\### (8) "SIGMA reveals subpopulations in a fetal human kidney data set". All the analyses in this section only support the accuracy of the clustering done by the authors, not the motivation for clustering given by SIGMA. So they do not really give any supporting evidence for SIGMA. In other words, another group of investigators can just perform these clustering practices and draw the same conclusion. There is no need for SIGMA here. This is in contrast to "SIGMA corresponded well with a visual inspection of the cluster UMAPs (Fig. 2a).", where the application in the other dataset does somewhat support the usefulness of SIGMA, though not in the strongest way, either.###

***We would like to stress that SIGMA is mainly meant as a tool to prioritize clusters for sub-clustering and validation of the resulting subpopulations. Indeed, anyone could have done the same clustering practices and probably would have obtained the same results, but they might have chosen different clusters to begin with and the subpopulations could have turned out to be purely driven by noise. For a complex data set with many clusters, it is impossible in practice to pursue all potential subpopulations with expensive and time-consuming experiments. SIGMA helps focusing precious resources on those clusters that are most likely to have meaningful (non-random) structure. We report the result on fetal human kidney to show that the clusters nominated by SIGMA indeed contained biologically interesting subpopulations.***

Minor comments:

###(1) "created new clusters as weighted averages" Meaning is not clear ###

***We would like to point the reviewer to the Methods section, where we describe the related procedure in detail:

“CD8 T cells and B cells were extracted from the data and each cluster was standardized gene-wise and cell-wise before the calculation of the singular value Powered by decomposition. To remove any sub-structure in these clusters and before the reconstruction of the matrices from the SVD, singular values above the MP distribution were moved into the bulk, and the transcriptome mode (i.e. the singular vector that would have the largest singular value without normalization, see Supplementary Methods Note 1) was moved above the MP distribution. Then, two synthetic clusters containing 150 cells each were created from the cleaned-up original clusters. For cluster 1, a weighted average of a randomly picked B cell with expression profile c_B and a randomly picked CD8 T cell with expression profile c_(CD8 T) was calculated according to: c_1= α∙c_B+(1- α)∙c_(CD8 T). For cluster 2, the weights were flipped: c_2= (1-α)∙c_B+α∙c_(CD8 T). α was chosen in a range from 0 to 1. α close to 0.5 produced highly similar clusters, while α close to 0 or 1 produced maximally different clusters (see Fig S3e). For each value of α, the procedure was repeated 50 times, each with a different seed for selecting 300 cells per cell type, and the results were averaged.”***

###(2) "As for BMNCs, SIGMA corresponded well with a qualitative assessment of cluster variability and G-SIGMA resulted in a similar ranking (Fig. S8a)." Not sure how this figure supports this claim. The authors need to explain the rationale in the main text. ###

*** We suspect that the reviewer’s question was related to a missing figure reference, which was added to the new version of the manuscript:

“As for BMNCs, SIGMA corresponded well with a qualitative assessment of cluster variability (Fig. 3a): Clusters with visible sub-clusters had the highest values of SIGMA. Ordering the clusters by G-SIGMA resulted in a similar ranking as SIGMA (Fig. S10a).”***

REVIEWER #3

\### 1) What are the advantages, if any, over the more standard approach where one first tries to extract low rank signals and then test whether these signals are significant compared to the estimated additive errors. ###

***To our knowledge, there are several approaches that fit the reviewer’s description. We were unsure which might be considered “standard”, so we picked three that we believe to be the most relevant: robust PCA, the dip test and ZINB-WaVE. We believe that SIGMA has multiple advantages over these methods: 1. It does not have any adjustable input parameters that have to be provided by the user or learned from the data. 2. It does not require the data to have a particular structure or the noise to follow a certain distribution. 3. It detects subtle phenotypes with higher sensitivity than other methods 4. Its values have a larger dynamic range and have a smaller uncertainty than other methods. 5. It is robust to over-clustering of the data, because clusters that only contain random noise and all sub-clusters of such a cluster will receive a SIGMA value of identically 0. To explain these advantages further, we added a detailed comparison between other methods and SIGMA to the discussion:

“There are several other methods that attempt to detect the presence of meaningful information in single-cell RNA-seq data. Below, we will compare SIGMA to some of the most popular examples: the silhouette coefficient, ROGUE, robust PCA, the dip test and ZINB-WaVE.

The silhouette coefficient is a popular tool to assess clustering quality. In contrast to SIGMA, this coefficient requires a (sub-)clustering and it cannot be used to decide, whether a cluster contains meaningful variability and should be sub-clustered further. As demonstrated, using the silhouette coefficient can lead to over-clustering of random noise as well as missing the presence of subtly different phenotypes. Likewise, SIGMA appeared to be more sensitive than ROGUE, an entropy-based clusterability measure. Both ROGUE and the silhouette coefficient do not scale linearly with the tARI, which we introduced as an upper limit to the achievable agreement of an empirical clustering with the ground truth.

Robust PCA [33,34] decomposes a measured expression matrix into a sparse component and a low-rank component. Under the assumption that noise is sparse, the sparse component is identified with random noise. In our opinion, there is no reason to assume that the noise in scRNA-seq data is sparse, or sparser than the measured expression matrix itself. Likely, every non-zero gene expression measurement was corrupted by noise. Additionally, the remaining low-rank component cannot be identified as the noise-free signal. It is fundamentally impossible to reconstruct the noise-free signal from the measured expression because the noise is created by a random process. The low-rank component is therefore only a (noisy) approximation of the noise-free signal. Given the fundamental limit to signal reconstruction, the best thing we can do is quantify the closeness between signal and measured expression, as implemented by SIGMA. In robust PCA, the low-rank matrix is often further subjected to dimensionality reduction, where it is difficult to determine the correct number of dimensions. SIGMA does not require any dimensionality reduction and uses all available data.

The dip test [7], a method aimed at detecting the presence of clusters, tests whether there are multiple modes in the data. It can be applied directly to the distribution of distances between expression profiles or a low-dimensional representation of the data, such as principal component scores. The dip test will miss relevant variability, if it does not manifest itself as separate modes, which can easily occur, for example in the case of differentiation paths. It also just provides a binary result (modes present or not), whereas SIGMA is a continuous measure and does not require the presence of modes.

ZINB-WaVE [26] performs dimensionality reduction based on a zero-inflated negative binomial distribution and is similar to principal component analysis, if no additional covariates are added to the model. ZINB-WaVE acknowledges the fact that principal components are prone to correlate with nuisance parameters, even after normalization. The problem is circumvented by adding such parameters as covariates to the model, which is similar to the confounder regression used for SIGMA. However, the user has to decide the number of dimensions to use and currently there is no principled way to determine the optimal number. SIGMA does not have any such adjustable parameters.”***

###2) It is not clear whether the SIGMA statistics is always related to clusterability. In the paper this is demonstrated by just one simulation example. Is it possible to come up with examples whether where there are non-Gaussian clusters that spread out in such as way they captured by a few singular directions. ###

***We are grateful for the reviewer’s suggestion to explore clusters with irregular shapes. Indeed, in developmental biology we often come across differentiation paths that appear as irregular continua of expression profiles in scRNA-seq data rather than compact clusters. In the new version of the manuscript, we show an example of differentiation paths in Fig. S5c. The reviewer is entirely correct in pointing out that such structures can lead to several relevant singular vectors (with outlying singular values). Depending on the biological question, this behavior could be useful or unwanted. One might actually want to separate the initial state of the differentiation (such as a stem cell state) from the differentiated state. In this case it is useful if SIGMA detects relevant substructure. Alternatively, one might consider a differentiation path as one cluster and might aim at separating it from other paths. In that case, we can apply trajectory inference and use the resulting pseudotime in the confounder regression step of SIGMA. In Fig. S5c we show that this results in individual differentiation paths having a corrected SIGMA of close to 0. In the new version of the manuscript, we describe these new results as follows:

“Confounder regression can also help to analyze data sets that are not made up of regular clusters but contain irregularly shaped continua of gene expression. For example, in developmental and stem cell biology we commonly observe differentiation paths, which are large clusters with gradually changing expression profiles. Uncorrected SIGMA values are high for such paths, which suggests meaningful subpopulations (S5c,d). Depending on the biological question, it might in fact be desirable to cluster differentiation paths, for example, to separate a stem cell state from a differentiated cell type. For other applications, it could be preferable to treat a differentiation path as one cluster. In that case we can use pseudotime approaches [28] to infer a temporal order of the gene expression profiles and use the inferred pseudotime in the confounder regression. If all observed variability is explained by developmental dynamics, SIGMA is reduced to 0 and thus no sub-clustering is suggested (S5c,d). “***

\### 3) The main use of this method is probably to replace silhouette scores. In figure S5, the silhouette scores seems to offer the same qualitative information as the new method. A more thorough comparison to silhouette scores should be conducted and discussed.###

***We agree with the reviewer, that the silhouette coefficient is one of the most popular methods to assess clustering quality. A more detailed comparison was indeed in order. Originally, we regarded the silhouette coefficient as a way to validate SIGMA and potentially quantify clusterability as defined in our manuscript (i.e., the best theoretically achievable agreement with a known ground truth). In the old Fig. S5 we first subdivided the data set into the clusters indicated in the figure and calculated the silhouette coefficient, separately for each cluster, on the ground truth clustering with Euclidean distances in PCA space. In the old Fig. S3, we use the best achievable clustering for the two clusters present in the simulated data as input to the silhouette coefficient to obtain tSIL. In Fig. S4 (previously Fig. S3), we continue to use the silhouette coefficient in this way and show that it is not ideal as quantification of our notion of clusterability. However, as the reviewer points out, the silhouette coefficient can also be seen as an alternative to SIGMA. In practice, the silhouette coefficient is used on clusterings of a complete data set to determine the overall clustering quality. Typically, multiple clusterings are created and the one with the highest silhouette coefficients is chosen. Therefore, we recalculated the silhouette coefficient in the new Fig. S6 (previously Fig. S5) in the following way: We use the entire data set with the indicated clustering as input to the silhouette coefficient, with Euclidean distances in PCA space. Then, we report average values per cluster.

To represent this approach to the silhouette coefficient, we added barplots of the silhouette coefficient for individual cells in Fig. S6 (bottom row). We also added an entirely new supplementary figure (Fig. S7) to compare the silhouette coefficient to SIGMA in multiple ways. In contrast to SIGMA, using the silhouette coefficient might cause the user to overlook subtly different phenotypes (Fig. S7a,b), as well as overcluster pure noise (Fig. S7e), depending on the situation. We discuss the comparison between SIGMA and the silhouette coefficient in this new paragraph:

“To compare SIGMA with the silhouette coefficient in more detail, we carried out additional simulations (Fig. S7). First, we simulated 3 clusters and subsequently merged two of them. While SIGMA clearly distinguished the merged cluster from the pure cluster, the silhouette coefficients were similar for both. Increasing the fraction of genes that are differentially expressed between the merged cluster increased the difference in silhouette coefficient, but only gradually (Fig. S7b). By contrast, SIGMA jumped to values close to 1 for the merged cluster for very small fractions of differentially expressed genes (around 0.03). It is therefore the more sensitive measure. The silhouette coefficient strongly depends on the number of principal components used in dimensionality reduction (Fig. S7c), as well as the metric for distances between expression profiles (Fig. S7d). SIGMA does not depend on such user-defined parameters. Most importantly, the silhouette coefficient cannot answer the question, whether an identified cluster contains meaningful substructure, as it requires partitioning into at least 2 sub-clusters. We simulated a cluster without any substructure and all variability was purely random (Fig. S7e). The silhouette coefficient was maximal for a k-means clustering with k=2, which might prompt a user to conclude (wrongly) that there are 2 sub-clusters present. SIGMA, which does not require any further partitioning of the cluster, was 0, indicating correctly that the observed variability was consistent with random noise. All in all, these comparisons indicate that SIGMA is a sensitive measure, which detects differences between highly similar phenotypes."***

REFERENCES

[1]Benaych-georges F, Rao R. The singular values and vectors of low rank perturbations of large rectangular random matrices. J Multivar Anal 2012;111:120–35. <https://doi.org/10.1016/j.jmva.2012.04.019>.

**2^nd^ Round**

**Reviewer 1**

In their paper, A clusterability measure for single-cell transcriptomics reveals phenotypic subpopulations, Mircea et al developed a new computational method and metric, SIGMA, for assessing the clustering performance on single-cell RNA-seq data-sets. By applying random matrix theory, the authors demonstrate that gene expression profiles can be split into separate noise and signal components, distinguished by the cut-off in a Marchenko-Pastur distribution. The authors apply this approach to several RNA-seq data-sets, identifying new populations within clusters and experimentally verifying this biology. I find that the authors did an excellent job in addressing my initial comments on this manuscript. The new version very nicely explains random matrix theory, and verifies several of the assumptions in their algorithm, and as such can act as a “tutorial” on how to apply RMT in high-throughput biological data. I was also interested in how reliable the underlying assumptions of the SIGMA approach are, and how one can derive modelling-like uncertainties on this method. The authors provide very good evaluations on both of these concerns, in quite some detail. The updated figures in the main text nicely reflect these changes, as do the substantial updates to the text covering an overview of RMT. Authors should address some minor editorial/grammar checks.

**Reviewer 2**

The reviewer has systematically addressed my concerns. Thank you

**Reviewer 3**

I am satisfied with the revision. The authors had addressed all my concerns.
